# Benchmarking of Oxford Nanopore whole genome sequencing for germline variant and CpG methylation detection across Canada’s national platform for genome sequencing and analysis

**DOI:** 10.3389/fgene.2026.1863034

**Published:** 2026-08-12

**Authors:** Jose Hector Galvez, Scott Mastromatteo, Kieran O’Neill, Robert Eveleigh, Haig Djambazian, Bhooma Thiruvahindrapuram, Eric Chuah, Shu-Huang Chen, Amirhossein Hajianpour, Zhuozhi Wang, Tara A. Paton, Sachin Desai, Sanjeev Pullenayegum, Lan He, Pawan Pandoh, Yongjun Zhao, Karen Mungall, Andrew J. Mungall, Richard F. Wintle, Guillaume Bourque, Stephen W. Scherer, Steven Jones, Mark Lathrop, Meredith McLaren, Jiannis Ragoussis

**Affiliations:** 1 McGill Genome Centre, Victor Phillip Dahdaleh Institute of Genomic Medicine, McGill University, Montreal, QC, Canada; 2 Canadian Centre for Computational Genomics, McGill University, Montreal, QC, Canada; 3 The Centre for Applied Genomics, The Hospital for Sick Children and University of Toronto, Toronto, ON, Canada; 4 Canada’s Michael Smith Genome Sciences Centre, BC Provincial Health Services Authority, Vancouver, BC, Canada; 5 Department of Human Genetics, McGill University, Montreal, QC, Canada; 6 CGEn – Canada’s National Platform for Genome Sequencing and Analysis, Toronto, ON, Canada

**Keywords:** benchmarking, long-reads sequencing, methylation, nanopore, whole genome sequencing

## Abstract

Advances in sequencing technology have enabled population-level Whole Genome Sequencing (WGS) efforts to be undertaken in many countries. Often, this requires collaboration across a distributed network of sequencing centres to allow efficient use of existing resources. Previously we tested the robustness of short-read sequencing technology and analysis pipelines across three established sequencing centres located in Montreal, Toronto, and Vancouver, constituting CGEn, Canada’s national platform for genome sequencing and analysis (www.cgen.ca). In this work, we extend the study to cover *Oxford Nanopore Technologies* (ONT) long read-based WGS technology which is increasingly being used for large-scale genomics studies. Thus, we performed ONT WGS of the HG002 cell line, a well-characterized standard obtained directly from the Coriell Institute, aiming for a minimum of 30× coverage using one R10.4 PromethION flowcell at each centre. The sequencing datasets were analyzed using commonly developed pipelines for SNVs, Indels, SV, and CpG methylation detection and then compared to the relevant publicly available GIAB benchmark datasets. As a result, we tested the robustness of the laboratory protocols as well as the effectiveness of the analytical pipelines for simultaneous analysis of genomic variation and CpG methylation. Key findings include: SNV detection with higher F1-scores in RefSeq Coding regions for the ONT datasets (99.1%–99.5%) compared to *Illumina* NovaSeqX data (96.5%); additionally, there was high correlation of CpG methylation across all the sequencing centres (R = 0.97), as well as with publicly available WGBS (R = 0.88) and EM-Seq (R = 0.93) data from the EpiQC study.

## 1 Introduction

In recent years, advances in long-read sequencing technologies have resulted in improvements in the length, accuracy, and cost-efficiency. Benchmarking studies of both instrument performance and analysis tools across various applications, from base calling focusing on raw signal-to-base conversion (Pagès-Gallego and De Ridder, 2023), variant detection (Hall et al., 2024), and genome assembly (Muñoz-Barrera et al., 2025), using standardized metrics and datasets, have been published. Key areas of comparison included the accuracy of different basecaller models, the effectiveness of bioinformatics tools for tasks like variant calling and assembly, and the comparison of *Oxford Nanopore Technologies* (ONT) data against other technologies like *Illumina* short reads. Benchmarking is crucial for standardizing evaluations and driving improvements in accuracy, especially as new hardware (e.g., R10.4) and software tools are released. Additionally, newer versions of long-read sequencers produce signal data that can be used to detect base modifications without requiring additional sequencing, a significant advantage that is increasingly relevant as multi-omics approaches gain momentum (Lee et al., 2020). Benchmarking and development of tools for CpG methylation (Yuen et al., 2021) has also demonstrated the potential of nanopore sequencing to detect base modifications and integrate with all advantages of long-read sequencing (O’Neill et al., 2024).

Large-scale sequencing efforts often require the pooling of resources across institutions to meet capacity demands, which is why in 2020, CGEn, Canada’s national platform for genome sequencing and analysis, underwent efforts to compare and benchmark short-reads (*Illumina*) sequencing performance across three sequencing centres in Vancouver, Toronto, and Montreal (Corbett et al., 2020). More recently, the increasing demand for long-read sequencing, as well as additional collaborative projects (such as the Canadian Precision Health Initiative [CPHI] by Genome Canada), have motivated a continuation of these benchmarking efforts using ONT’s PromethION platform. The benchmarking to be presented here focuses on the comparison of DNA library preparation protocols and the performance of PromethION flowcells across the three sites. The sequencing data processing and analysis against known truth sets was done centrally and focused on detection of both germline genome variation as well as base-modification.

To facilitate comparison across centres, as well as with previously generated and published data, we used the son (HG002/NA24385) sample in the Genome in a Bottle (GIAB) Consortium Ashkenazim Father-Mother-Son trio (Zook et al., 2016) for this benchmarking.

## 2 Materials and methods

### 2.1 Sample

The sample sequenced by all centres corresponds to the National Institute of Standards and Technology (NIST) reference material 8,392. Described as the son in the family trio of Eastern Europe Ashkenazi Jewish Ancestry (HG002/GM24385/NA24385) (Zook et al., 2016) and a part of the Genome in a Bottle (GIAB) Consortium.

Homogenized growths of the same B lymphoblastoid HG002 cell line from the Human Genetic Cell Repository at Coriell Institute for Medical Research were shipped to each CGEn sequencing centre (Canada’s Michael Smith Genome Sciences Centre in Vancouver, BC [**BCGSC**]; The McGill Genome Centre in Montreal, QC [**McGill**]; and The Centre for Applied Genomics at SickKids in Toronto, ON [**TCAG/SickKids**]) for independent DNA extraction and library preparation.

### 2.2 Oxford nanopore library preparation

Extraction of genomic DNA (gDNA) and sequencing library preparation was carried out independently at each sequencing centre using the following specifications. All sites used a *Qiagen* MagAttract HMW DNA extraction Kit. Shearing of gDNA was performed at the BCGSC using g-TUBE (*Covaris*) centrifugation [2800 rpm-2pass], an instrument of the same brand was used at SickKids [2800 rpm-2pass, 3000 rpm-2pass]. McGill performed shearing using a Megaruptor [40 ng/μL, 100 μL, speed: 31]. Size selection at BCGSC and SickKids was performed using a Blue Pippin (*Sage Science*) – 10 kb High Pass cassette, whereas McGill used SRE XS 10 kb. The ONT libraries were prepared at all centres following the instructions of a standard ONT kit with catalogue identifier SQK-LSK114.

### 2.3 Oxford nanopore sequencing and basecalling

Sequencing and basecalling were performed independently at each sequencing centre using the following specifications. Libraries were loaded into a single ONT R10.4.1 flowcell (FLO-PRO114M) with a minimum of 6,500 available pores. The flowcell was then sequenced using an ONT PromethION 48 (PRO-SEQ048) instrument running the MinKNOW [v24.06.10] on-board software. After sequencing, the raw signal data was basecalled using the Dorado Basecall Server [v7.4.12] tool (which contains functionality and models from the standalone Dorado v0.6.3) and the “Super-accurate basecalling model” (v4.3.0, 400 bps), with modified basecalling parameter turned on (modifications 5hmC and 5mC, all contexts).

### 2.4 *Illumina* library preparation and sequencing

For short-read sequencing, gDNA was extracted from the same cell line directly using the SageHLS High Molecular Weight DNA Library System (*Sage Science, Inc.*, Beverly, Massachusetts, United States). The quality of the extracted gDNA was assessed using an *Agilent* Tapestation and quantified using the Qubit DNA High Sensitivity (HS) assay. The library was prepared using the *Illumina* TruSeq PCR-Free Library Prep Kit, with 500 ng of high-quality gDNA as input. DNA was fragmented to a target size of 300bp using the Covaris LE220 focused ultrasonicator (*PerkinElmer*), followed by size selection with Ampure XP beads (*Beckman Coulter*). Sequencing was carried out on the *Illumina* NovaSeqX platform with paired-end 150bp reads (2 × 150) on one lane with 1% PhiX as an internal control.

### 2.5 Bioinformatics analyses

#### 2.5.1 Short-read alignment using BWA-MEM2

The NovaSeqX sample was processed using GenPipes v6.1.0 (Bourgey et al., 2019), a bioinformatics pipeline based on GATK Best Practices. Briefly, raw reads were aligned to GRCh38. p14 human reference using BWA-MEM2 v.2.2.1 (Md et al., 2019). Post-alignment processing using GATK mark duplicates v4.6.0.0 (Poplin et al., 2017) identifies and marks both PCR and optical duplicates for exclusion in downstream variant calling.

#### 2.5.2 Long-read alignment using minimap2

After basecalling, long-read sequencing data was aligned to the GRCh38.p14 human reference using minimap2 v.2.28 (Li, 2021). The raw BAM files were converted to FASTQ prior to alignment using samtools v1.19.2 (Danecek et al., 2021), with the option to include the base modification tags (MM,ML) in the FASTQ header. When aligning, the ‘map-ont’ preset was selected in minimap2 to ensure optimal performance for aligning ONT data to the reference. The Z-drop score and inversion Z-drop score were also modified to [600,200] for all samples. Finally, the `-y` flag was also selected so that the ‘MM,ML’ tag information was copied from the headers of the FASTQ files and into the new alignment BAMs. After alignment, the files were then sorted and indexed with samtools v1.19.2.

Basic metrics and plots were generated after alignment using nanoplot v1.42.0 (De Coster and Rademakers, 2023), mosdepth v0.3.4 (Pedersen and Quinlan, 2018) and multiQC v1.25.1 (Ewels et al., 2016) with their default settings.

#### 2.5.3 Running Clair3 for small variant detection

The aligned long-reads of each sample were analyzed with Clair3 v1.0.10 (Lin et al., 2022) to detect germline variants, specifically SNVs and Indels. The tool was run in parallel, by chromosome, using the model ‘**r1041_e82_400bps_sup_v430**’ for the long-reads as well as the ‘longphase’ module turned on to enable phasing of the detected variants. The aligned short-reads were similarly analyzed using DeepVaraint v1.9.0 (Poplin et al., 2018). Afterwards, individual chromosome VCF files were merged and indexed using bcftools v1.15 (Danecek et al., 2021). The phasing information was used to generate haplo-tagged BAM files for all ONT datasets using WhatsHap v2.5 (Martin et al., 2016).

#### 2.5.4 Germline small variant assessment

The precision, sensitivity, and F1-scores of germline SNP and Indel calls were evaluated using the hap. py v0.3.15 (Zook et al., 2016) algorithm and RTG vcfeval v3.12.1 (Cleary et al., 2015). Variant calls from each centre were compared against the Telomere-2-Telomere (T2T-Q100 v1.1) and Challenging Medically Relevant Genes (CMRG) v1.0.0 truth sets for HG002 (Jarvis et al., 2022; Wagner et al., 2022). Default parameters were used for CMRG, with T2T having the additional argument of `–gender male’ set to correctly call chromosome X as suggested by GIAB authors. This analysis provided a comprehensive assessment of variant detection accuracy across technologies, including comparisons of individual variant callers.

#### 2.5.5 Structural variant detection with long-reads data

Structural Variant (SV) detection using ONT long-reads was performed using three separate tools, each implementing a different algorithm. Dysgu v1.8.9 (Cleal and Baird, 2022), Sniffles2 v2.8.0 (Smolka et al., 2024), svim v2.0.0 (Heller and Vingron, 2019) and cuteSV v2.1.3 (Jiang et al., 2020) were all run with default parameters and a minimum SV size threshold of 30bp to enable detection across a wide range of SV classes, including insertions, deletions, inversions, duplications and translocations. Because of differences in the reporting format utilized for some SV classes across algorithms, only deletions and insertions were extracted and systematically compared. However, known complex structural alterations in HG002 (Wagner et al., 2022) were manually verified using the alignment files and inspecting the output of each SV caller.

#### 2.5.6 Structural variant benchmarking

The performance of SV detection was benchmarked using the truvari v4.3.1 (English et al., 2022) on the two truth sets: the truth set for genome-wide assessment T2T and the SVs from the CMRG dataset. The T2T truth set included 20,767 deletions and 29,640 insertions, all over 30 bp in length and the CMRG subset encompassed 89 high-quality deletions and 105 insertions located near 273 known clinical genes. Benchmarking metrics, including precision, recall, and F1-scores, were generated for each variant file from various sequencing technologies, which were processed by bcftools v2.30 (Danecek et al., 2021) to retain SVs with the caller specific ‘PASS FILTER’ with alternative contigs removed. Truvari assessed the filtered variant using the bench arguments ‘--sizemin 50 –sizefilt 30 –sizemax 150k -r 2000 -C 5000 --pctseq 0 --pick ac--dup-to-ins’ with the refine arguments of ‘--recount--use-region-coords--use-original-vcfs--align mafft’ applied to only the T2T set.

Systematic comparison of complex SV events beyond insertions and deletions is complicated by differences in how these events are reported by the different callers, as well as a reduced number of observable events in HG002. However, to explore the ability of long-reads sequencing to detect some of these complex events, we manually inspected the VCFs and alignment files to verify a known variant in the GPI gene in this sample, which consists of an insertion in an intronic Variable Number of Tandem Repeat (VNRT) region (Wagner et al., 2022).

#### 2.5.7 Downsampling of data

The long- and short-reads data produced by all centres was randomly downsampled to 30×, 20×, 10× and 5× depth of coverage to assess the impact of lower coverage on the results. The downsampling was done using the rasusa v2.1.0 (Hall, 2022) tool, which accounts for read length, for long reads in particular, as part of its downsampling calculation. Downsampling was repeated 10 times with a different seed for each sample at each depth, to account for any variation in the random sampling. After downsampling, samples were processed in the same way as the full-depth samples, as described above.

#### 2.5.8 Construction and benchmarking of genome variation graphs

*De novo* genome assembly was performed independently for ONT long-read data generated at each of the three sites using hifiasm v0.25.0-r726 (Cheng et al., 2021). Reads passing quality filters (PASS) were assembled in ONT mode, which leverages long-read overlaps to construct an assembly graph and resolve haplotypes where possible. Telomeric sequences were preserved by specifying the human telomere motif (“CCCTAA”) using the ‘--telo-m’ option; all other parameters were set at their default values. Contigs were screened for the DNA control strand (3.6 kb amplicon mapping to the 3′ end of the lambda genome). No partial matches were detected, and contigs with full-length matches were removed from the assembly. Assembly quality was evaluated using standard metrics, including total assembly length, contig LG50 and NG50, and contig count.

Assemblies were aligned to the paternal HG002 Q100 (plus maternal chromosome X) assembly using MashMap (Kille et al., 2023) [‘--pi 85 --blockLength 100000 -f one-to-one’] to detect misassemblies and split contigs prior to graph construction. A variation graph was created using cactus-pangenome v3.0.1 (Hickey et al., 2024) and parameter `--mgSplit’ from two reference assemblies (CHM13v2.0, GRCh38) and ten haploid assemblies of HG002: HG002 Q100 v1.1 (paternal, maternal), a PacBio + HiC assembly, BCGSC, McGill and SickKids assemblies.

The tool vg deconstruct v1.69.0 (Liao et al., 2023) using parameters ‘--all-snarls -L 0.90’ was run to detect variants and produce a VCF with calls relative to GRCh38. Multiallelic sites were decomposed using bcftools norm v1.20 (‘-m both’), vcfbub v0.1.0 decomposed complex bubbles into primitive alleles (maximum size of 100 kb), and we used vcfwave (vcflib v1.0.13) to harmonize allele representation (Danecek et al., 2021; Garrison et al., 2022).

Structural variant calls from the assemblies were evaluated using Truvari v5.4.0 (English et al., 2022) following using the same procedure described above for structural variant benchmarking. The tool odgi untangle (Guarracino et al., 2022) was used to slice the graph and calculate identity between samples and HG002 Q100, which was required to create the karyoploteR images.

#### 2.5.9 Base modification detection and cross-technology concordance analysis

Base modifications (5mC and 5hmC) were extracted from the basecalled, haplotagged bams using ONT modkit v0.5.0 pileup.

For pairwise correlation analyses, pileups were extracted for all CpG sites. Pairwise Pearson correlations were carried out using the base R ‘cor ()’ function. For comparison to the EpiQC datasets (Foox et al., 2021), methylation bedgraph files were downloaded from GEO (accession GSE186383): WGBS (GSM5649436, GSM5649437), EMSeq (GSM5649517, GSM5649518). The ONT R9 chemistry replicates were obtained from the EpiQC authors, as the bedgraphs were missing from GEO. Downsampled comparisons were performed using the downsampled BAM files generated previously. For 5hmC, comparisons used 5hmC pileups at CpG sites. All plots were made using R.

For the imprinting analysis, we used a set of known imprinted DMRs, as described in (Akbari et al., 2023) and available at github.com/vahidAK/PatMat/blob/main/patmat/Imprinted_DMR_List_V1.GRCh38.tsv. Average methylation was computed for each haplotype at each iDMR using modkit pileup. Differential methylation was called on a per-iDMR basis using modkit dmr. All plots were made using R.

## 3 Results

### 3.1 Raw sequencing output was similar across the sites

The three CGEn sites (BCGSC, McGill, and SickKids) each received one aliquot of the same HG002/NA24385 cell line (i.e., the son in the GIAB Ashkenazim Father-Mother-Son trio). The gDNA was extracted and sequencing libraries were prepared independently at each site. T he ONT PromethION instrument available at each site was used to sequence the library prepared at their facility using only one flowcell per library (see materials and methods for additional details).

#### 3.1.1 Coverage metrics and alignment performance

**FIGURE 1:**
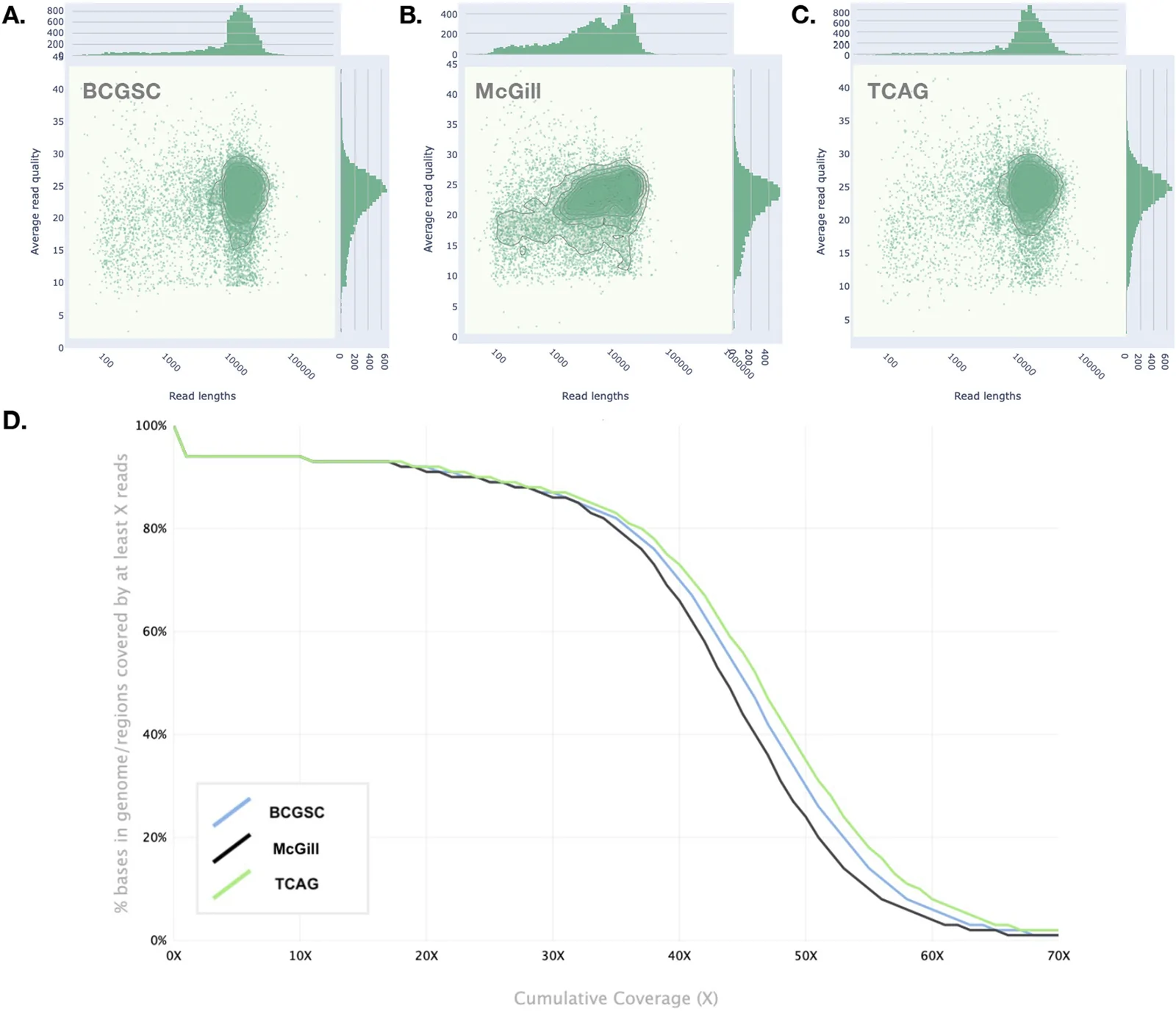
Summary Statistics of the Sequencing Output Across Sites. Kernel Density Estimate (KDE) plots showing the raw read lengths (histogram in the top) versus average read quality (histogram to the right side), which demonstrate the distribution of the raw data from: **(A)** BCGSC, **(B)** McGill Genome Centre, and **(C)** TCAG (SickKids). **(D)** Coverage distribution across three sites.

All sites achieved a mean depth of coverage above 40× using a single R10.4.1 flowcell with a minimum of 6,500 available pores. Top-level results for all three sites are summarized in Table 1 and Figure 1. Reads from BCGSC and SickKids, which used the same BP 10 kb HP size selection method, had similar average length of 15.4 kb and 17.0 kb, respectively. Whereas reads from McGill, which used SRE XS 10 kb for size selection, averaged 9.7 kb length. However, the McGill flowcell generated approximately 5M more reads than the other two, resulting in comparable coverage.

**TABLE 1:** Summary of sequencing statistics across sites.

Site	Mean Coverage [ × ]	Aligned Reads [M]	Mean Align. Len. [kb]	Med. Align. Len. [kb]	Med. Align. Qual	Align. N50 [kb]	Tot. Align. Bases [Gb]
*BCGSC*	44.3	9.2	15.4	14.8	23.5	19.9	138.2
*McGill*	43.1	14.7	9.7	6.1	22.5	18.8	134.5
*SickKids*	45.9	9.2	17.0	15.5	24.2	21.5	143.5

Reads were aligned to the GRCh38 reference (see materials and methods), and the resulting aligned read quality across the three sites was very similar, with a median Q-score of 23.5 [BCGSC], 22.5 [McGill] and 24.2 [SickKids]. The total amount of aligned bases was also comparable across sites: 138.2 Gb (44.3×) [BCGSC], 134.5 Gb (43.1×) [McGill] and 143.5 Gb (45.9×) [SickKids]. Coverage was evenly distributed across chromosomes in all cases.

### 3.2 Germline variant detection was comparable across sites

#### 3.2.1 SNV and indel variant detection results

**FIGURE 2:**
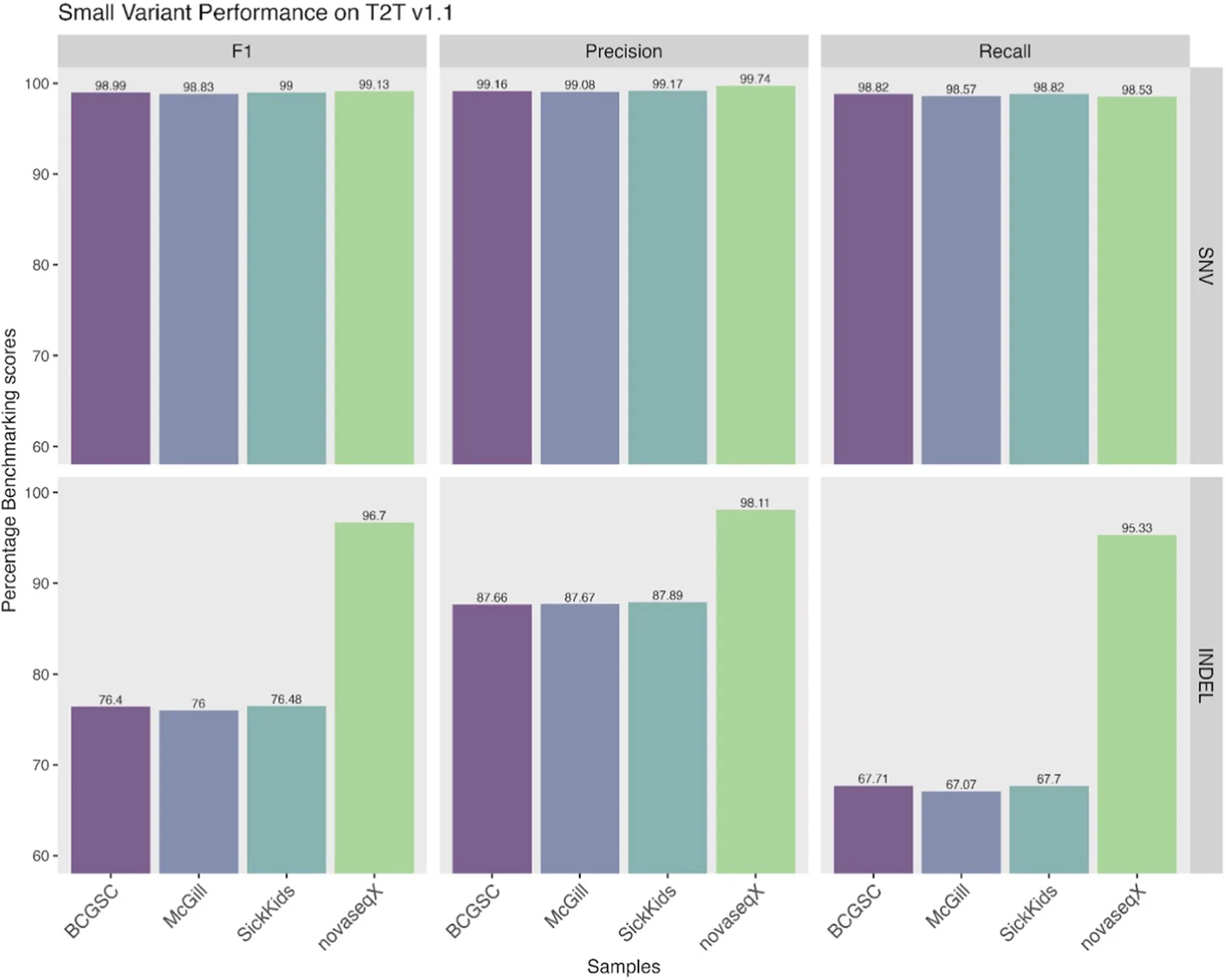
Germline Variant Detection F1, Precision, and Recall Scores. Plot comparing the SNV (top row) and Indel (bottom row) calls to the T2T v1.1, [which includes the complete human genome] truth set and the resulting F1, Precision, and Recall scores for the three ONT datasets, as well as the results of an *Illumina* NovaSeqX dataset of the same cell-line sequenced at mean depth of 46.4× and 315.8 bp mean insert size.

Small variant detection (SNV and Indels) was performed using Clair3 (Yu et al., 2026). The results of small variant calling were very similar across the three sites, with minimal differences in F1 score (Figure 2), when compared to the Telomere-to-Telomere (T2T) QV100 v1.1 small variant truth set as a point of reference (Jarvis et al., 2022; Hansen et al., 2025). The T2T truth set spans the whole human genome (including highly repetitive and challenging regions) and is the most complete truth set released to date for this dataset. Additionally, as a reference, variant calls were compared with those generated from the same sample using a short-read sequencer (*Illumina* NovaSeqX) at a similar depth of coverage (46.4×).

SNV detection was broadly comparable across platforms, but clear differences emerged between the ONT datasets (BCGSC, McGill, SickKids) and NovaSeqX. The ONT datasets consistently achieved higher recall (98.57%–98.82%) compared to NovaSeqX (98.53%), indicating improved sensitivity and fewer missed true positives. In contrast, NovaSeqX exhibited substantially higher precision (99.74% vs. 99.08%–99.17% for ONT), reflecting a lower false positive rate. Despite the recall advantage of ONT, the gain in precision for NovaSeqX had a larger impact on the overall F1 score, resulting in the highest F1 for NovaSeqX (99.13%) compared to slightly lower values for SickKids (99.00%), BCGSC (98.99%), and McGill (98.83%). Similar patterns (Supplementary Figure S1) were observed for SNVs in Challenging and Medically Relevant Genes (CMRG) (Wagner et al., 2022).

In contrast, Indel detection showed a markedly different pattern compared to SNVs, with a clear performance separation between NovaSeqX and the ONT datasets. The short-reads dataset substantially outperformed all ONT datasets, achieving both higher precision (98.11% vs. ∼87.66–87.89% for ONT) and higher recall (95.33% vs. ∼67.07–67.70%). This indicates that NovaSeqX not only produced far fewer false positives but also recovered a significantly larger proportion of true Indels. When comparing Indels in the CMRG truth set (Supplementary Figure S1), a similar pattern emerges with the NovaSeqX greatly outperforming ONT, although the long-reads datasets display a substantial improvement in recall (∼74.98–75.92%) which is also reflected in higher F1 scores.

In order to investigate the nature of the differences in the performance, we segregated both SNV and Indel calls by the genomic context (i.e., difficult, RefSeq coding, homopolymers, etc.) using the classification developed by (Jarvis et al., 2022). This analysis revealed a similar performance across the board for both SNVs and Indels, with ONT slightly outperforming compared to the NovaSeqX in the SNVs found on RefSeq coding regions (Supplementary Figure S2). However, there was a large difference between the short-reads and long-reads results in homopolymer regions greater than 12bp, where short-reads greatly outperform nanopore long-reads. This difference is even more pronounced in the longer homopolymer regions (greater than 21bp in length)**,** highlighting the need for further algorithm development to accurately detect variants in this type of genomic segments using long-reads. These results are expected and reflect technical biases in ONT basecalling especially in homopolymers.

#### 3.2.2 SV detection results

**FIGURE 3:**
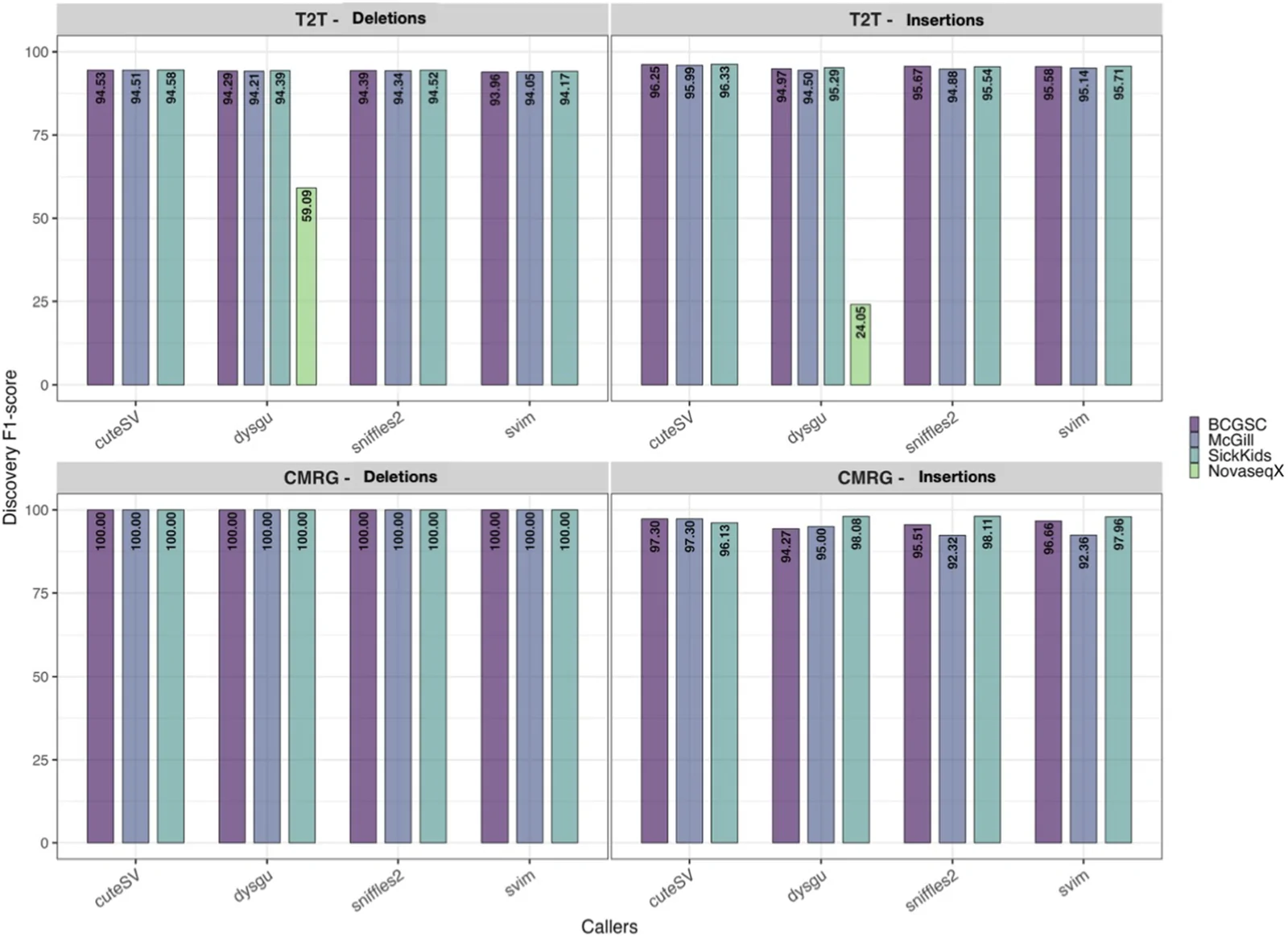
Structural Variant Detection F1 Scores. Plot comparing the F1 score for large deletions and large insertions (>50 bp) between the three sites using four different SV calling algorithms (dysgu, sniffles2, svim, and cuteSV). The top panel compares the SV caller results against the T2T SV truth set [which includes the complete human genome], whereas the bottom panel compares them against the CMRG SV truth set [which is a selection of clinically relevant genes].

Structural variants (SVs; variants of size >50bp) were detected using four different algorithms: dysgu (Cleal and Baird, 2022), sniffles2 (Smolka et al., 2024), svim (Heller and Vingron, 2019), and cuteSV (Jiang et al., 2020). These calls were compared to the T2T truth set (Jarvis et al., 2022; Hansen et al., 2025) as well as the CMRG SV truth set (Wagner et al., 2022) to determine the F1, precision, and recall scores. The results of the SV calling tools using the ONT long-reads were comparable across the three sites (Figure 3), with differences in performance mostly due to the algorithm choice, although some variation seems to be correlated to the sequencing site, specifically for CMRG events where we observe the SickKids sample overperforms with three of the four SV callers. Nonetheless, the differences between sites remain relatively minor, generally speaking.

When compared to the full T2T SV truth set, the F1 scores for large insertions and deletions were very similar across all sites and callers and above 94%. In general, all the long-read samples performed much better than the short-reads dataset, which only achieved an F1 score of ∼59% for deletions and >21% for insertions using dysgu, the only one of the three callers that is able to call SVs using short-reads. This result is expected since short-read insert sizes are generally much smaller than the average long-read, limiting the ability to detect larger events.

There is a greater variation across algorithms and samples when comparing the results to the CMGR truth set, with the dysgu caller performing the best for both SV types, especially in the BCGSC and TCAG samples. However, all long-reads samples and callers achieved F1 scores above 89.8 across when compared to this reduced truth set. A full breakdown of the precision, recall and F1 scores across samples, variant callers, and sequencing depths can be found in Supplementary Table S1.

One of the advantages of long-reads sequencing technologies is their ability to resolve complex structural events that are otherwise difficult to detect with short-reads technologies. However, systematic comparisons of complex SV variant calls are difficult because each caller reports complex events in slightly different ways. Additionally, there are only a few known events in HG002 that would be available for validation in this case. We manually inspected the alignments of all long-reads samples to verify the presence of the known 16,946 bp insertion in an intronic VNTR in the gene GPI (Wagner et al., 2022). We observe the insertion in all the samples, and notably, when separating the reads by haplotags it is clear that the event is only present in one of the haplotypes (Supplementary Figure S3).

### 3.3 *De novo* assembly and graph-based variant detection results

Samples from the three centres were assembled *de novo* with hifiasm (Cheng et al., 2021). Assemblies from all centres exhibited similar overall statistics including contiguity and completeness metrics. Additional QUAST-derived assembly metrics are summarized in Supplementary Table S2.

**FIGURE 4:**
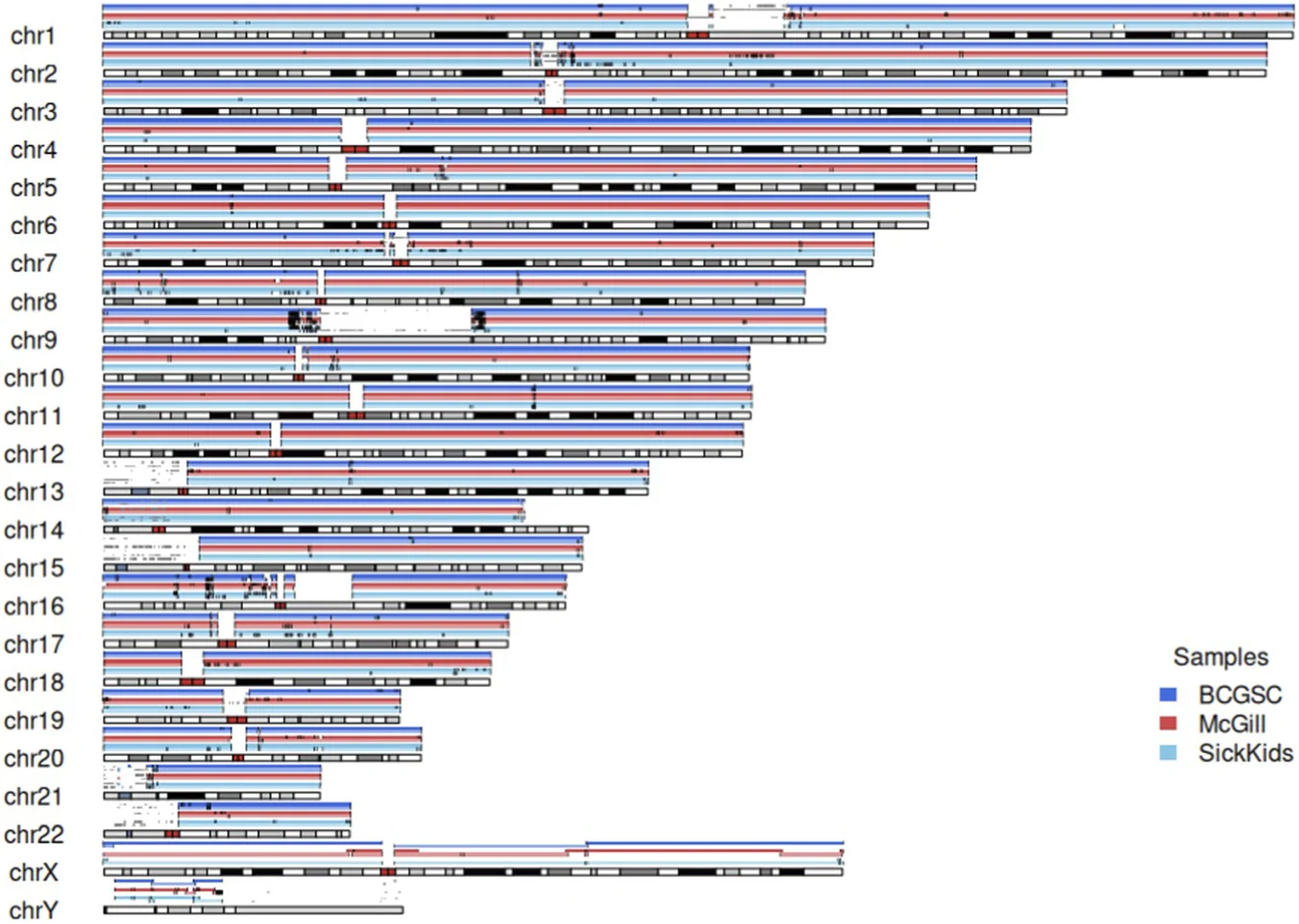
Visualization of Contigs from *De Novo* Assemblies. The plot shows the *de novo* haploid assemblies for all centres using the karyoploteR library. All assemblies have long continuous contigs spanning large portions of the genome. However, the vertical black lines designate contig discontinuity. These discontinuities and gaps cluster around centromeres, acrocentric, pseudo autosomal (which explains the large gap in the Y chromosome) as well as other high-complexity regions. Grey lines represent contigs that were discarded during graph construction due to being ambiguous.

Assemblies were combined into a HG002-specific variation graph to enable comparison to HG002 Q100 and easy mapping onto the CHM13 (T2T) and GRCh38 reference assemblies. This also enables VCF decomposition from a unified representation, improving consistency in calls which is reflected in the benchmarking consistency (Table 2). Projection of the assemblies onto the T2T reference shows they have highly consistent discontinuities, which can be observed as gaps in the visualization of the contigs presented in Figure 4. These gaps generally occur near centromeres, acrocentric, pseudo autosomal (explaining the large gap in the Y chromosome), and other high-complexity regions.

**TABLE 2:** Summary of graph-based SV detection results.

Value	SV Type	HG002 Q100	BCGSC	McGill	SickKids	PacBio + HiC
*Precision*	Del	96.89	96.72	96.69	96.68	96.60
*Recall*	Del	96.01	95.83	95.83	95.66	95.98
*F1*	Del	96.45	96.27	96.25	96.17	96.28
*Precision*	Ins	98.62	98.47	98.45	98.56	98.44
*Recall*	Ins	98.34	98.17	98.13	98.08	98.03
*F1*	Ins	98.48	98.32	98.29	98.32	98.23

**FIGURE 5:**
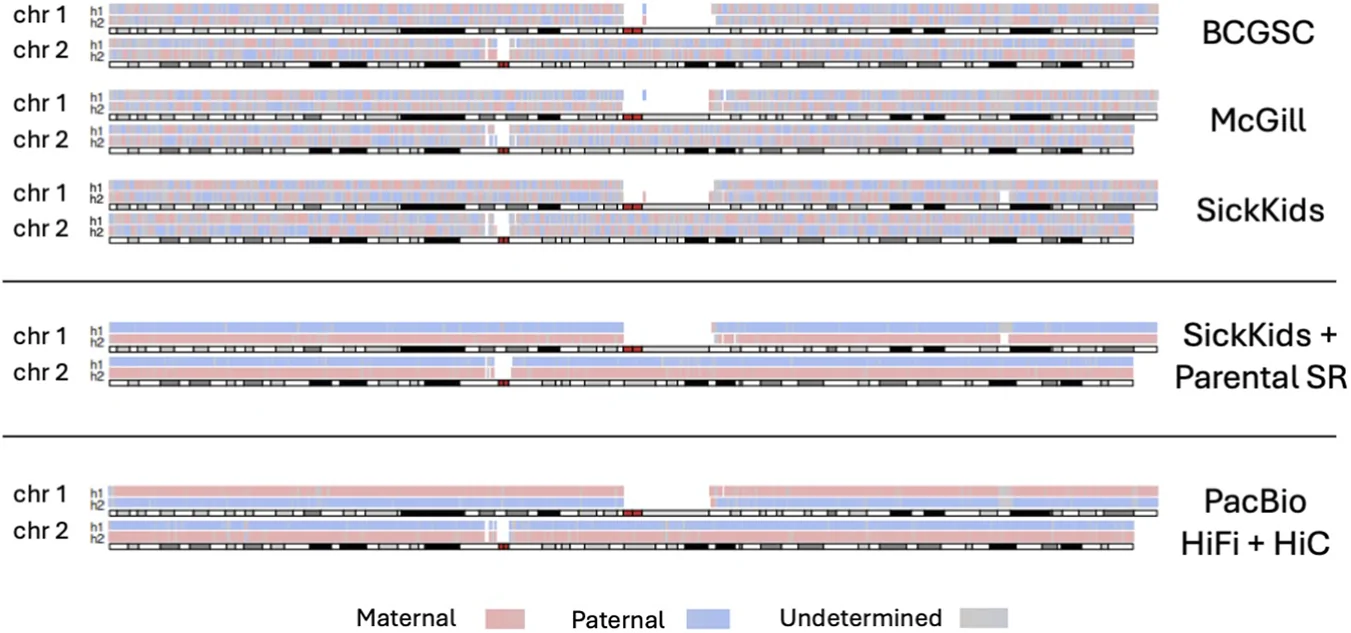
Phasing Block Comparison Across the *De Novo* Assemblies. The *de novo* assembled genomes were split into 100 kb segments and compared to both paternal and maternal HG002 Q100 sequences and then plotted using the karyoploteR library. Greater similarity to paternal haplotype is colored as blue and pink for maternal. A sliding window of 500 kb was used to smooth the plot, with windows colored as grey if it was not possible to assign them to either parental haplotype. In the top panel, the first two chromosomes of the ONT assemblies contain a high degree of phase switching between haplotype 1 (h1) and haplotype 2 (h2). The addition of parental short-reads data to the ONT data during the assembly greatly reduces phase switching (middle panel, showing the result for the SickKids sample only) and produces a similar result to the assembly generated using publicly available PacBio HiFi and HiC data (bottom panel).

We also utilized the variation graph to compare whether a section of an assembly was more similar to the maternal or paternal Q100 haplotypes, and visualized each contig using 500 kb windows where each window was colored based on whether it was more similar to one parental line or grey if it was not possible to assign (Figure 5; Supplementary Figure S4). Assemblies using only ONT data show a large number of phase switches, however we have found this can be corrected by including parental short-read data during hifiasm assembly (Figure 5). Supplementing the ONT data with parental short-read data produces similar results to assemblies using *PacBio* HiFi and HiC data.

### 3.4 Downsampling has major impacts on germline indel detection

To evaluate the potential impact of lower coverage, the long-read sequencing data for each centre was down sampled to 30×, 20×, 10×, and 5× depth of coverage using the tool rasusa (Hall, 2022), which in addition to taking into account sequencing depth also considers read-length to ensure a similar distribution for lower depths of coverage. The downsampling was repeated 10 times at each depth, to account for the effect of different random seeds. After downsampling, Clair3 was run on all datasets using the same parameters as the samples at full depth and the results were compared to the same truth sets. The results of this comparison are summarized in Table 3 and Supplementary Figures S5, S6.

**TABLE 3:** Summary of downsampling SNV/indel results.

Centre	Num. of Randomized Replicates	Coverage [ × ]	Variant Type	Precision		Recall		F1	
				*Mean*	*Std. Deviation*	*Mean*	*Std. Deviation*	*Mean*	*Std. Deviation*
BCGSC	10	5	SNV	86.34%	0.00024	92.62%	0.00014	89.37%	0.00017
BCGSC	10	10	SNV	96.74%	0.00010	97.97%	0.00009	97.35%	0.00008
BCGSC	10	20	SNV	98.83%	0.00009	98.72%	0.00005	98.78%	0.00005
BCGSC	10	30	SNV	99.12%	0.00005	98.70%	0.00004	98.91%	0.00004
BCGSC	1 (Full Depth)	44	SNV	99.16%	-	98.82%	-	98.99%	-
McGill	10	5	SNV	86.12%	0.00023	91.95%	0.00012	88.94%	0.00017
McGill	10	10	SNV	96.43%	0.00021	97.72%	0.00004	97.07%	0.00011
McGill	10	20	SNV	98.82%	0.00005	98.47%	0.00004	98.65%	0.00003
McGill	10	30	SNV	99.13%	0.00008	98.42%	0.00004	98.77%	0.00004
McGill	1 (Full Depth)	43	SNV	99.08%	-	98.57%	-	98.83%	-
SickKids	10	5	SNV	86.08%	0.00022	91.38%	0.00017	88.65%	0.00012
SickKids	10	10	SNV	96.78%	0.00015	97.78%	0.00012	97.28%	0.00010
SickKids	10	20	SNV	98.95%	0.00006	98.63%	0.00007	98.79%	0.00004
SickKids	10	30	SNV	99.19%	0.00004	98.68%	0.00004	98.94%	0.00003
SickKids	1 (Full Depth)	46	SNV	99.17%	-	98.82%	-	99.00%	-
*NovaseqX*	*10*	*5*	*SNV*	*96.82%*	*0.00009*	*87.11%*	*0.00012*	*91.71%*	*0.00008*
*NovaseqX*	*10*	*10*	*SNV*	*99.05%*	*0.00003*	*96.69%*	*0.00008*	*97.86%*	*0.00005*
*NovaseqX*	*10*	*20*	*SNV*	*99.56%*	*0.00002*	*98.39%*	*0.00002*	*98.97%*	*0.00002*
*NovaseqX*	*10*	*30*	*SNV*	*99.67%*	*0.00001*	*98.50%*	*0.00001*	*99.08%*	*0.00001*
*NovaseqX*	*1 (Full Depth)*	*47*	*SNV*	*99.74%*	*-*	*98.53%*	*-*	*99.13%*	*-*
BCGSC	10	5	INDEL	59.29%	0.00019	44.43%	0.00010	50.79%	0.00013
BCGSC	10	10	INDEL	74.48%	0.00022	56.16%	0.00018	64.04%	0.00016
BCGSC	10	20	INDEL	85.00%	0.00027	62.53%	0.00011	72.06%	0.00014
BCGSC	10	30	INDEL	87.25%	0.00010	65.32%	0.00011	74.71%	0.00009
BCGSC	1 (Full Depth)	44	INDEL	87.66%	-	67.71%	-	76.40%	-
McGill	10	5	INDEL	59.85%	0.00029	43.69%	0.00022	50.51%	0.00023
McGill	10	10	INDEL	74.39%	0.00014	55.70%	0.00015	63.70%	0.00011
McGill	10	20	INDEL	85.16%	0.00022	62.13%	0.00010	71.84%	0.00013
McGill	10	30	INDEL	87.32%	0.00012	64.96%	0.00011	74.50%	0.00009
McGill	1 (Full Depth)	43	INDEL	87.67%	-	67.07%	-	76.00%	-
SickKids	10	5	INDEL	59.72%	0.00040	43.20%	0.00024	50.13%	0.00027
SickKids	10	10	INDEL	74.54%	0.00026	55.61%	0.00016	63.70%	0.00019
SickKids	10	20	INDEL	85.34%	0.00022	61.94%	0.00010	71.79%	0.00013
SickKids	10	30	INDEL	87.46%	0.00011	64.94%	0.00009	74.54%	0.00008
SickKids	1 (Full Depth)	46	INDEL	87.89%	-	67.70%	-	76.48%	-
*NovaseqX*	*10*	*5*	*INDEL*	*87.31%*	*0.00043*	*69.63%*	*0.00033*	*77.47%*	*0.00034*
*NovaseqX*	*10*	*10*	*INDEL*	*93.32%*	*0.00016*	*86.08%*	*0.00025*	*89.56%*	*0.00018*
*NovaseqX*	*10*	*20*	*INDEL*	*97.11%*	*0.00008*	*93.78%*	*0.00006*	*95.42%*	*0.00006*
*NovaseqX*	*10*	*30*	*INDEL*	*97.84%*	*0.00003*	*94.90%*	*0.00006*	*96.35%*	*0.00004*
*NovaseqX*	1 (Full Depth)	*47*	*INDEL*	*98.11%*	-	*95.33%*	-	*96.70%*	-

Overall, downsampling has a larger effect on the detection of Indels than SNVs. In the data from all three centres, there was minimal impact (<0.2%) in the F1 score for SNV detection when downsampling to 20×. Even at 5× depth, all three centres still achieved an F1 score >93.0% when compared to the SNV truth set. These results are consistent with equivalent tests performed on *Illumina* short-read data at 5× depth of coverage, where the F1 score for SNV detection is slightly below 90% (Eveleigh et al., 2026). The similarity of performance between short and long reads at lower depths of coverage opens up the possibility of variant imputation using similar methods to those developed for *Illumina* short-reads. However, a full comparison of variant imputation was out-of-scope for this study.

Conversely, Indel detection is more directly affected by downsampling, mostly due to impacts in the recall score (i.e., there were more missed true positives with each decrease in coverage). However, precision also decreases considerably (to below 90%) in most cases when the coverage is below 20×, indicating that in addition to missing true positives, there are also more false positives at lower depths of coverage.

### 3.5 Germline variant phasing

Germline variants were assigned to phase blocks as part of the Clair3 workflow using the longphase tool (Lin et al., 2022). At full coverage, all samples achieved a phase block NG50 higher than 600 kb (Table 4). The samples from BCGSC and SickKids each produced around 5,800 blocks with an NG50 of approximately 870 kb, whereas the McGill sample had slightly more blocks (8,253) and a lower NG50. This difference can be attributed to the smaller average read size in the McGill sample compared to the other two.

**TABLE 4:** Summary of phasing results.

Sample	Num. of Randomized Replicates	Coverage [×]	Phase Blocks (Mean)	Phase Blocks (S.d.)	Switch Errors (Mean)	Switch Errors (S.d.)	Flip Errors (Mean)	Flip Errors (S.d.)	Phase Block NG50 (Mean)	Phase Block NG50 (S.d.)
BCGSC	10	5	16,244.8	49	3,506.3	35	377.8	8	315,752.5	3,952
BCGSC	10	10	13,227.3	36	830.7	11	133.8	8	435,605.7	2,882
BCGSC	10	20	7,944.0	7	250.6	9	30.4	4	688,054.7	1,234
BCGSC	10	30	6,703.0	5	174.2	6	22.6	2	811,135.7	2051
BCGSC	1 (Full Depth)	44	5,921.0	-	167.0	-	17	-	914,780.0	-
McGill	10	5	20,043.2	60	4,097.1	26	482.7	11	246,731.5	8,410
McGill	10	10	15,982.8	52	1,070.7	20	187.8	15	351,928.5	1752
McGill	10	20	10,167.4	17	245.2	7	41	2	532,986.3	1,014
McGill	10	30	8,919.4	9	184.6	5	38.2	3	615,593.3	1,447
McGill	1 (Full Depth)	43	8,327.0	-	177.0	-	25	-	667,684.0	-
SickKids	10	5	16,139.3	61	3,912.6	42	358.1	16	327,014.7	7,759
SickKids	10	10	13,204.2	27	816.1	9	115.7	8	441,525.9	1,622
SickKids	10	20	7,965.2	10	217.0	8	26.2	3	679,496.8	2,991
SickKids	10	30	6,721.3	12	175.4	7	26	3	799,463.8	1956
SickKids	1 (Full Depth)	46	5,862.0	-	146.0	-	20	-	923,335.0	-

Despite the minor differences in the number of phasing blocks and their NG50, all centres had similar switch and flip errors at full depth, with the McGill sample having the lowest number of errors, which can also be a result of the lower NG50 at >40× depth. When downsampling, the number of phase blocks increases proportionally to the decrease in coverage, whereas the NG50 values behave inversely. In other words, less depth of coverage results in smaller contigs, on average, which is expected. The switch and flip errors, however, remain relatively stable even when downsampling to 20× depth, and only show a marked increase in error rates when coverage is at 10× or 5×.

### 3.6 Base modification results are highly correlated across sites

#### 3.6.1 CpG detection results

**FIGURE 6:**
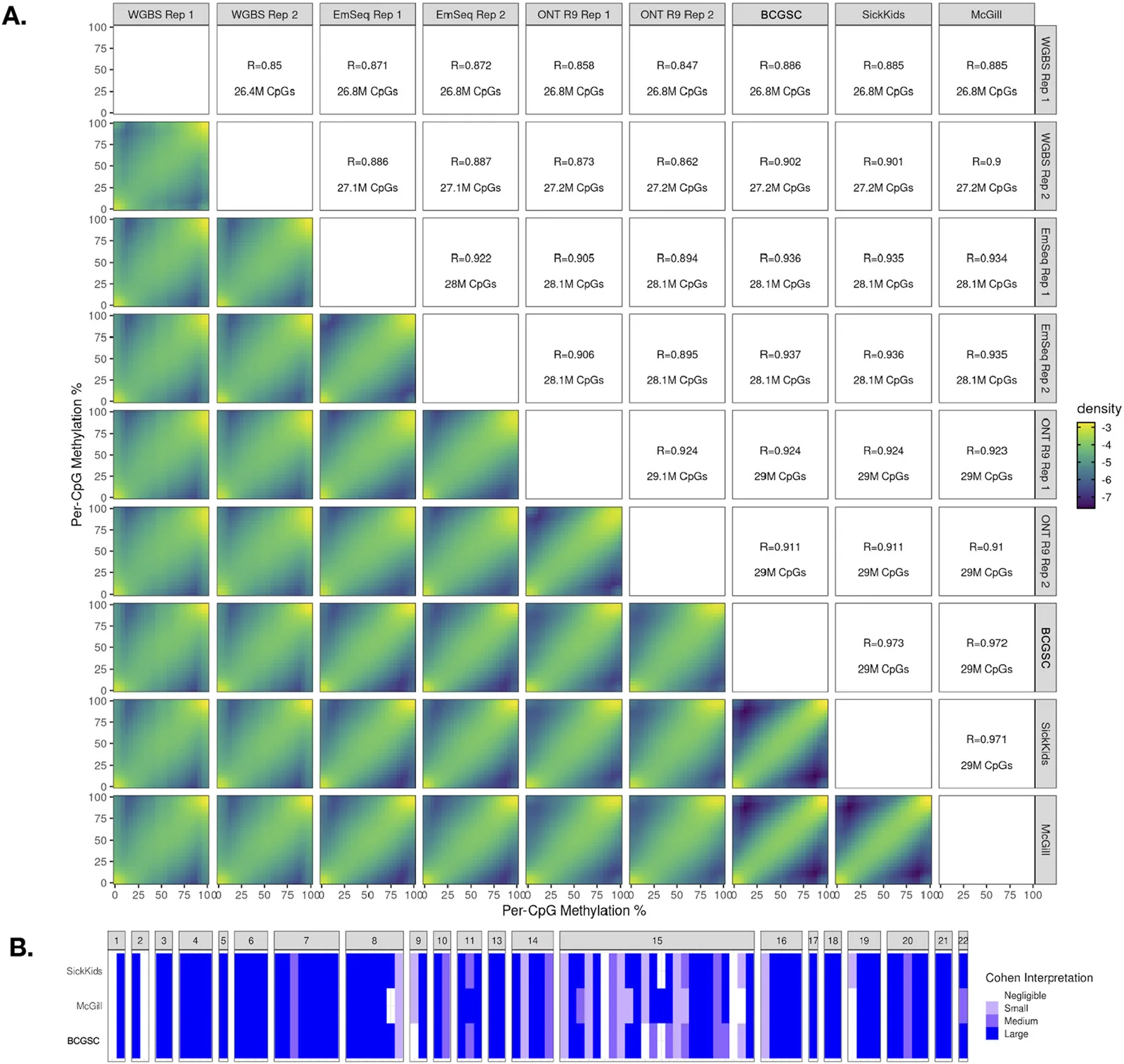
DNA Methylation Results Compared Across Sites. **(A)** Correlation matrix of the methylation percentage at each CpG site, with data derived from the EpiQC study, including two WGBS replicates, two EM-Seq replicates and two ONT R9 replicates, against the data from the three CGEn sites (ONT R10.4). Correlation coefficients are higher for the three ONT R10.4 datasets. The number of CpG sites interrogated is included in each box. **(B)** A plot showing the interpretation of Cohen’s H score of the effect size of the difference in methylation between haplotypes across each dataset and all autosomal chromosomes (large: h>=0.8; medium: 0.8 > h >=0.5; small: 0.5 > h >=0.2). The effect sizes are mostly consistent in all the datasets.

Base modifications were extracted from the bam files using the ONT tool ‘modkit’ v0.5.0. We performed a cross-technology comparison of the nanopore datasets produced for this study with data generated by the SEQC2 epigenomics quality control (EpiQC) study (Foox et al., 2021). This included whole-genome bisulfite (WGBS) and enzymatic modification (EM-seq) short-read *Illumina* sequencing data, as well as R9 chemistry ONT data. Correlation of per-CpG fractional methylation and overlap in terms of the number of CpGs covered were calculated between each pair (Figure 6A). Correlation of the detected CpG methylation was higher between the three CGEn sites (R = 0.971–0.972) than between any other combination of technologies. The data from the three sites also showed the greatest overlap of number of CpGs covered, at 29.1M. Of the EpiQC datasets, the EM-seq showed the closest correlation to the cross-centre data, while the R9 ONT data showed the closest overlap in terms of number of CpGs covered (at 29.1M).

This correlation showed only modest degradation in response to downsampling (Supplementary Figure S7), continuing to outperform all other modalities at 20× coverage, and to outperform WGBS even as low as 10×. This suggests potential utility for ONT sequencing as a more accurate replacement for modalities such as WGBS, with potential cost savings due to this accuracy being maintained even at low-pass coverage.

#### 3.6.2 Phased methylation analysis

Long-read sequencing technologies have the unique ability to enable phasing across kilobases to megabases, while simultaneously reading out base modifications from the same sequencing reads. This enables phasing of methylation data across functional regions, and from functional regions to their targets. To assess the repeatability of this phasing across centres, we examined a set of imprinting regions compiled from five studies (Akbari et al., 2023), keeping only those regions reported in two or more studies. Imprinting regions (iDMRs) are expected to show differential methylation between haplotypes across human individuals and tissues and form a useful metric for assessing methylation phasing.

We used modkit v0.5.0 to extract phased methylation calls from the haplotagged bam files and then ran DMR calling between the iDMRs (by calculating Cohen’s h). The difference in the effect size in the iDMRs is shown in Figure 6B and Supplementary Figure S8, categorized by the interpretation criteria set out by Cohen (Cohen, 2013)), specifically large: h>=0.8; medium: 0.8 > h >=0.5; small: 0.5 > h >=0.2. While some iDMRs showed small or negligible difference in methylation between haplotypes, this was consistent across centres, suggesting that it is the result of the underlying biology of the sample. The vast majority of iDMRs were consistently called as differentially methylated in data from all three centres. This capability of accurately resolving phased methylation is unique to long-read sequencing, and has potential clinical applications, such as detecting MSH2 promoter methylation in Lynch syndrome (O’Neill et al., 2024).

#### 3.6.3 Detection of 5hmC

Nanopore sequencing enables the detection of 5-hydroxymethylcystosine (5hmC) in addition to the more common 5-methylcytosine. We assessed the correlation between centres using the same methodology as for 5mC, the results of which are shown in Supplementary Figure S9. Correlations were much lower than for 5 mC (0.382–0.394), but still non-negligible. This is likely due to 5hmC being a much rarer mark than 5mC, and as such, distinguishing signal from noise remains challenging with current tools.

## 4 Discussion

The work presented here underlines the robustness of HMW DNA extraction, ONT library preparation and flowcell performances across different CGEn sites. Minor differences in the library preparation process at each sequencing centre notwithstanding, the output for all metrics and variant types was highly comparable across sites. This was achieved despite minor differences in DNA shearing and small fragment elimination protocols that were applied at the library preparation stage in different centres. The two datasets derived from g-tube shearing methods showed a slightly larger sequencing read length N50 resulting in slight improvements in variant calling performance when directly comparing F1-scores. We are aware that one of the limitations of this study is that it has only focused on comparing a single, well-characterized sample, but we believe that this initial benchmark provides the groundwork for further standardization that is required for larger initiatives.

Interestingly, our results also show that the performance of SNV and Indel detection of the ONT platform against the established *Illumina* standard is now on par in the RefSeq coding regions of the human genome. However, it still lags on longer homopolymers (>11 nucleotides), which continues to be an area of intense technical development at ONT. Conversely, the nanopore reads were better suited for structural variant detection than short-reads, as expected. Here, short-reads may narrow the gap once graph-based pangenome references are more commonly utilized. One of the most promising aspects of long-reads technologies is their ability to resolve complex structural events. We show that in this sample, we can detect an insertion in an intronic VNTR in the gene GPI, a known event in HG002. However, the reduced number of true complex events in this sample available for validation is a limitation of this study and additional characterization of long-reads performance for the detection of complex SVs is something we will explore in the future with more suitable examples.

Random downsampling of the datasets to depths of 30×, 20×, 10×, and 5×, show similar effects across the board. For SNVs, the F1-scores remain relatively stable even at depths of 20× with significant loss of sensitivity at 10× and even more so at 5× depth. However, Indel detection is always impacted by lower coverage, proportionally to the loss of depth. These results are expected and similar to what has been reported for *Illumina* short-reads, which opens the door to potential variant imputation at lower depths, a technique that is now common with short reads, and which will be explored further in future studies.

The samples from all three sites yielded similar results when used to generate *de novo* assemblies with hifiasm. Projection of these assemblies to the T2T reference indicates that discontinuities in the assembly were also consistent across sites and more common near centromeres, acrocentric, pseudo autosomal, and other high-complexity regions. Phasing of SNV and Indels was also highly comparable across sites, with the McGill sample producing a lower NG50 likely due to the shorter average read length.

Base modifications results, specifically CpG methylation, were also highly correlated across centres, which is encouraging for future applications that want to include these results in their WGS analyses. Our benchmarking also revealed a high correlation between WGBS, EM-seq, and ONT data for CpG methylation detection. Detection of modified bases remained highly correlated even at lower depths of coverage, outperforming the WGBS benchmark at depths of coverage as low as 10×, indicating that ONT is a highly suitable alternative for this kind of analysis. Similarly, the phasing of modified bases and iDMRs detection was highly consistent across centres, with minor differences in the effect size in some regions (see Figure 6B). The ability to accurately resolve phased methylation is unique to long-read sequencing, and has potential clinical applications, making this one of the most exciting applications of this technology.

Joint benchmarking and comparison efforts across sequencing centres in Canada improve the feasibility of large-scale projects across the region. Already, efforts such as the Canadian Biogenome Project and Genome Canada’s Canadian Precision Health Initiative (CPHI) projects are allowing researchers to distribute samples to their nearest facility for convenience and cost reduction. Although the results presented in this study represent a “best-case” scenario, and only encompass a single standardized sample, they suggest that r egardless of the CGEn site that produced it, ONT data is highly comparable. There are additional factors to consider when designing cross-centre initiatives, including availability of local computational resources to run the analyses, however for the purposes of this study, it was assumed that every site has the technical resources available perform the analysis as described above and an evaluation of computational resources and runtime was out-of-scope.

In the near future, this will prove crucial for upcoming high-profile genomics projects in Canada that will partner with CGEn for data production (e.g., CPHI, and Terry Fox Research Institute’s Marathon of Hope, among others). It also showcases the feasibility for not only national, but also large-scale international projects to be carried out across sites utilizing ONT technology; including initiatives such as the international T2T (https://www.genome.gov/about-genomics/telomere-to-telomere) and RNAome projects (https://humanrnomeproject.org/).

## Data Availability

The datasets generated for this study can be found in the European Nucleotide Archive (ENA) study accession PRJEB111322 (https://www.ebi.ac.uk/ena/browser/view/PRJEB111322). The code used to run the analyses can be found in the following GitHub repository: github.com/c3g/cgen_ont_benchmark.

## References

[B1] Akbari V. Hanlon V. C. T. O'Neill K. Lefebvre L. Schrader K. A. Lansdorp P. M. (2023). Parent-of-origin detection and chromosome-scale haplotyping using long-read DNA methylation sequencing and Strand-seq. Cell. Genomics 3 (1), 100233. 10.1016/j.xgen.2022.100233 36777186 PMC9903809

[B2] Bourgey M. Dali R. Eveleigh R. Chen K. C. Letourneau L. Fillon J. (2019). GenPipes: an open-source framework for distributed and scalable genomic analyses. GigaScience 8 (6), giz037. 10.1093/gigascience/giz037 31185495 PMC6559338

[B3] Cheng H. Concepcion G. T. Feng X. Zhang H. Li H. (2021). Haplotype-resolved *de novo* assembly using phased assembly graphs with hifiasm. Nat. Methods 18 (2), 170–175. 10.1038/s41592-020-01056-5 33526886 PMC7961889

[B4] Cleal K. Baird D. M. (2022). Dysgu: efficient structural variant calling using short or long reads. Nucleic Acids Res. 50 (9), e53. 10.1093/nar/gkac039 35100420 PMC9122538

[B5] Cleary J. G. Braithwaite R. Gaastra K. Hilbush B. S. Inglis S. Irvine S. A. (2015). Comparing variant call files for performance benchmarking of next-generation sequencing variant calling pipelines. Bioinformatics. 10.1101/023754

[B6] Cohen J. (2013). Statistical Power Analysis for the Behavioral Sciences. 2nd Edition. New York: Routledge. 10.4324/9780203771587

[B7] Corbett R. D. Eveleigh R. Whitney J. Barai N. Bourgey M. Chuah E. (2020). A distributed whole genome sequencing benchmark study. Front. Genet. 11, 612515. 10.3389/fgene.2020.612515 33335541 PMC7736078

[B8] Danecek P. Bonfield J. K. Liddle J. Marshall J. Ohan V. Pollard M. O. (2021). Twelve years of SAMtools and BCFtools. GigaScience 10 (2), giab008. 10.1093/gigascience/giab008 33590861 PMC7931819

[B9] De Coster W. Rademakers R. (2023). NanoPack2: population-scale evaluation of long-read sequencing data. Bioinformatics 39, btad311. 10.1093/bioinformatics/btad311 37171891 PMC10196664

[B10] English A. C. Menon V. K. Gibbs R. A. Metcalf G. A. Sedlazeck F. J. (2022). Truvari: refined structural variant comparison preserves allelic diversity. Genome Biol. 23 (1), 271. 10.1186/s13059-022-02840-6 36575487 PMC9793516

[B11] Eveleigh R. J. M. Reiling S. J. Galvez J. H. Bourgey M. Ragoussis J. Bourque G. (2026). Benchmarking of sequencing technologies defines optimal strategies for genetic variants detection in a human genome. Genome Biol. 27 (129), 129. 10.1186/s13059-026-04048-4 41952156 PMC13072666

[B12] Ewels P. Magnusson M. Lundin S. Käller M. (2016). MultiQC: summarize analysis results for multiple tools and samples in a single report. Bioinformatics 32 (19), 3047–3048. 10.1093/bioinformatics/btw354 27312411 PMC5039924

[B13] Foox J. Nordlund J. Lalancette C. Gong T. Lacey M. Lent S. (2021). The SEQC2 epigenomics quality control (EpiQC) study. Genome Biol. 22 (1), 332. 10.1186/s13059-021-02529-2 34872606 PMC8650396

[B14] Garrison E. Kronenberg Z. N. Dawson E. T. Pedersen B. S. Prins P. (2022). “A spectrum of free software tools for processing the VCF variant call format: vcflib, bio-vcf, cyvcf2, hts-nim and slivar. PLOS Comput. Biol. 18 (5), e1009123. 10.1371/journal.pcbi.1009123 35639788 PMC9286226

[B15] Guarracino A. Heumos S. Nahnsen S. Prins P. Garrison E. (2022). ODGI: understanding pangenome graphs. Bioinformatics 38, 3319–3326. 10.1093/bioinformatics/btac308 35552372 PMC9237687

[B16] Hall M. (2022). Rasusa: randomly subsample sequencing reads to a specified coverage. J. Open Source Softw. 7 (69), 3941. 10.21105/joss.03941

[B17] Hall M. B. Wick R. R. Judd L. M. Nguyen A. N. Steinig E. J. Xie O. (2024). Benchmarking reveals superiority of deep learning variant callers on bacterial nanopore sequence data. eLife 13, RP98300. 10.7554/eLife.98300 39388235 PMC11466455

[B18] Hansen N. F. Dwarshuis N. Ji H. J. Rhie A. Loucks H. Logsdon G. A. (2025). A complete diploid human genome benchmark for personalized genomics. bioRxiv. 10.1101/2025.09.21.677443 42561913 PMC13456413

[B19] Heller D. Vingron M. (2019). SVIM: structural variant identification using mapped long reads. Bioinformatics 35, 2907–2915. 10.1093/bioinformatics/btz041 30668829 PMC6735718

[B20] Hickey G. Monlong J. Ebler J. Novak A. M. Eizenga J. M. Gao Y. (2024). Pangenome graph construction from genome alignments with minigraph-cactus. Nat. Biotechnol. 42 (4), 663–673. 10.1038/s41587-023-01793-w 37165083 PMC10638906

[B21] Jarvis E. D. Formenti G. Rhie A. Guarracino A. Yang C. Wood J. (2022). Semi-automated assembly of high-quality diploid human reference genomes. Nature 611 (7936), 519–531. 10.1038/s41586-022-05325-5 36261518 PMC9668749

[B22] Jiang T. Liu Y. Jiang Y. Li J. Gao Y. Cui Z. (2020). Long-read-based human genomic structural variation detection with cuteSV. Genome Biol. 21 (1), 189. 10.1186/s13059-020-02107-y 32746918 PMC7477834

[B23] Kille B. Garrison E. Treangen T. J. Phillippy A. M. (2023). “Minmers are a generalization of minimizers that enable unbiased local jaccard estimation. Bioinformatics 39, btad512. 10.1093/bioinformatics/btad512 37603771 PMC10505501

[B24] Lee I. Razaghi R. Gilpatrick T. Molnar M. Gershman A. Sadowski N. (2020). Simultaneous profiling of chromatin accessibility and methylation on human cell lines with nanopore sequencing. Nat. Methods 17 (12), 1191–1199. 10.1038/s41592-020-01000-7 33230324 PMC7704922

[B25] Li H. (2021). New strategies to improve minimap2 alignment accuracy. Bioinformatics 37, 4572–4574. 10.1093/bioinformatics/btab705 34623391 PMC8652018

[B26] Liao W.-W. Asri M. Ebler J. Doerr D. Haukness M. Hickey G. (2023). A draft human pangenome reference. Nature 617 (7960), 312–324. 10.1038/s41586-023-05896-x 37165242 PMC10172123

[B27] Lin J.-H. Chen L. C. Yu S. C. Huang Y. T. (2022). LongPhase: an ultra-fast chromosome-scale phasing algorithm for small and large variants. Bioinformatics 38, 1816–1822. 10.1093/bioinformatics/btac058 35104333

[B28] Martin M. Patterson M. Garg S. O Fischer S. Pisanti N. Klau G. W. (2016). WhatsHap: fast and accurate read-based phasing. Bioinformatics. 10.1101/085050

[B36] Md V. Misra S. Li H. Aluru S. (2019). “Efficient architecture-aware acceleration of BWA-MEM for multicore systems,” in 2019 IEEE International Parallel and Distributed Processing Symposium (IPDPS). 2019 IEEE International Parallel and Distributed Processing Symposium (IPDPS) (Rio de Janeiro, Brazil: IEEE), 314–324. 10.1109/IPDPS.2019.00041

[B29] Muñoz-Barrera A. Rubio-Rodríguez L. A. Jáspez D. Corrales A. Marcelino-Rodriguez I. Ortiz L. (2025). Benchmarking of bioinformatics tools for the hybrid *de novo* assembly of human and non-human whole-genome sequencing data. Comput. Struct. Biotechnol. J. 27, 3099–3109. 10.1016/j.csbj.2025.07.020 40703096 PMC12284544

[B30] O’Neill K. Pleasance E. Fan J. Akbari V. Chang G. Dixon K. (2024). Long-read sequencing of an advanced cancer cohort resolves rearrangements, unravels haplotypes, and reveals methylation landscapes. Cell. Genomics 4 (11), 100674. 10.1016/j.xgen.2024.100674 39406235 PMC11605692

[B31] Pagès-Gallego M. De Ridder J. (2023). Comprehensive benchmark and architectural analysis of deep learning models for nanopore sequencing basecalling. Genome Biol. 24 (1), 71. 10.1186/s13059-023-02903-2 37041647 PMC10088207

[B32] Pedersen B. S. Quinlan A. R. (2018). Mosdepth: quick coverage calculation for genomes and exomes. Bioinformatics 34, 867–868. 10.1093/bioinformatics/btx699 29096012 PMC6030888

[B33] Poplin R. Ruano-Rubio V. DePristo M. A. Fennell T. J. Carneiro M. O. Van der Auwera G. A. (2017). Scaling accurate genetic variant discovery to tens of thousands of samples. Genomics. 10.1101/201178

[B34] Poplin R. Chang P. C. Alexander D. Schwartz S. Colthurst T. Ku A. (2018). A universal SNP and small-indel variant caller using deep neural networks. Nat. Biotechnol. 36 (10), 983–987. 10.1038/nbt.4235 30247488

[B35] Smolka M. Paulin L. F. Grochowski C. M. Horner D. W. Mahmoud M. Behera S. (2024). Detection of mosaic and population-level structural variants with Sniffles2. Nat. Biotechnol. 42 (10), 1571–1580. 10.1038/s41587-023-02024-y 38168980 PMC11217151

[B37] Wagner J. Olson N. D. Harris L. McDaniel J. Cheng H. Fungtammasan A. (2022). Curated variation benchmarks for challenging medically relevant autosomal genes. Nat. Biotechnol. 40 (5), 672–680. 10.1038/s41587-021-01158-1 35132260 PMC9117392

[B38] Yu X. Zheng Z. Chen L. Qin Z. He M. Luo R. (2026). Leveraging ONT move table values for signal aware variant calling. bioRxiv. 10.64898/2026.02.13.705285 PMC13501324 42635225

[B39] Yuen Z.W.-S. Srivastava A. Daniel R. McNevin D. Jack C. Eyras E. (2021). Systematic benchmarking of tools for CpG methylation detection from nanopore sequencing. Nat. Commun. 12 (1), 3438. 10.1038/s41467-021-23778-6 34103501 PMC8187371

[B40] Zook J. M. Catoe D. McDaniel J. Vang L. Spies N. Sidow A. (2016). Extensive sequencing of seven human genomes to characterize benchmark reference materials. Sci. Data 3 (1), 160025. 10.1038/sdata.2016.25 27271295 PMC4896128

