# Supplementary material for: Benchmarking of Oxford Nanopore whole genome sequencing for germline variant and CpG methylation detection across Canada’s national platform for genome sequencing and analysis
Source: Front Genet. 2026 Aug 12;17:1863034. doi: 10.3389/fgene.2026.1863034 (PMC13506141; doi:10.3389/fgene.2026.1863034)
Supplement: Supplementary file 1 [file Supplementaryfile1.docx]

Supplementary Material

# 1 Supplementary Figures and Tables

## 1.1 Supplementary Tables

**Supplementary Table 1: SV Detection Performance by Algorithm and Depth of Coverage**

| **Sample** | **Truth Set** | **SV Type** | **Coverage** | **Num. of Replicates*** | **SV Caller** | **Mean**  **F1 Score** | **Median**  **F1 Score** | **Std. Dev. F1 Score** |
| --- | --- | --- | --- | --- | --- | --- | --- | --- |
| BCGSC | T2T | insertion | 5 | 1 | cuteSV | 0.0% | 0.0% | NA |
| BCGSC | T2T | deletion | 5 | 10 | dysgu | 82.0% | 82.1% | 0.00135 |
| BCGSC | T2T | insertion | 5 | 10 | dysgu | 86.6% | 86.7% | 0.00157 |
| BCGSC | T2T | deletion | 5 | 10 | sniffles2 | 74.5% | 74.4% | 0.00213 |
| BCGSC | T2T | insertion | 5 | 10 | sniffles2 | 76.4% | 76.4% | 0.00170 |
| BCGSC | T2T | deletion | 5 | 10 | svim | 80.3% | 80.3% | 0.00064 |
| BCGSC | T2T | insertion | 5 | 10 | svim | 85.8% | 85.8% | 0.00133 |
| BCGSC | T2T | deletion | 10 | 10 | cuteSV | 51.2% | 51.2% | 0.00083 |
| BCGSC | T2T | insertion | 10 | 10 | cuteSV | 53.5% | 53.6% | 0.00147 |
| BCGSC | T2T | deletion | 10 | 10 | dysgu | 92.2% | 92.2% | 0.00086 |
| BCGSC | T2T | insertion | 10 | 10 | dysgu | 94.7% | 94.7% | 0.00061 |
| BCGSC | T2T | deletion | 10 | 10 | sniffles2 | 92.3% | 92.3% | 0.00065 |
| BCGSC | T2T | insertion | 10 | 10 | sniffles2 | 93.3% | 93.2% | 0.00067 |
| BCGSC | T2T | deletion | 10 | 10 | svim | 93.5% | 93.5% | 0.00044 |
| BCGSC | T2T | insertion | 10 | 10 | svim | 95.2% | 95.2% | 0.00068 |
| BCGSC | T2T | deletion | 20 | 10 | cuteSV | 83.9% | 83.9% | 0.00085 |
| BCGSC | T2T | insertion | 20 | 10 | cuteSV | 88.0% | 88.0% | 0.00073 |
| BCGSC | T2T | deletion | 20 | 10 | dysgu | 93.9% | 93.9% | 0.00063 |
| BCGSC | T2T | insertion | 20 | 10 | dysgu | 95.3% | 95.3% | 0.00045 |
| BCGSC | T2T | deletion | 20 | 10 | sniffles2 | 94.2% | 94.2% | 0.00029 |
| BCGSC | T2T | insertion | 20 | 10 | sniffles2 | 95.5% | 95.5% | 0.00071 |
| BCGSC | T2T | deletion | 20 | 10 | svim | 95.0% | 95.1% | 0.00108 |
| BCGSC | T2T | insertion | 20 | 10 | svim | 96.4% | 96.4% | 0.00070 |
| BCGSC | T2T | deletion | 30 | 10 | cuteSV | 94.1% | 94.1% | 0.00027 |
| BCGSC | T2T | insertion | 30 | 10 | cuteSV | 95.7% | 95.7% | 0.00024 |
| BCGSC | T2T | deletion | 30 | 10 | dysgu | 94.3% | 94.3% | 0.00066 |
| BCGSC | T2T | insertion | 30 | 10 | dysgu | 95.3% | 95.3% | 0.00067 |
| BCGSC | T2T | deletion | 30 | 10 | sniffles2 | 94.5% | 94.5% | 0.00040 |
| BCGSC | T2T | insertion | 30 | 10 | sniffles2 | 95.9% | 95.9% | 0.00038 |
| BCGSC | T2T | deletion | 30 | 10 | svim | 95.0% | 95.0% | 0.00103 |
| BCGSC | T2T | insertion | 30 | 10 | svim | 96.7% | 96.7% | 0.00069 |
| McGill | T2T | deletion | 5 | 10 | dysgu | 81.2% | 81.2% | 0.00139 |
| McGill | T2T | insertion | 5 | 10 | dysgu | 85.3% | 85.3% | 0.00085 |
| McGill | T2T | deletion | 5 | 10 | sniffles2 | 74.3% | 74.3% | 0.00107 |
| McGill | T2T | insertion | 5 | 10 | sniffles2 | 74.5% | 74.5% | 0.00180 |
| McGill | T2T | deletion | 5 | 10 | svim | 80.1% | 80.0% | 0.00237 |
| McGill | T2T | insertion | 5 | 10 | svim | 84.0% | 84.1% | 0.00259 |
| McGill | T2T | deletion | 10 | 10 | cuteSV | 50.1% | 50.2% | 0.00083 |
| McGill | T2T | insertion | 10 | 10 | cuteSV | 46.8% | 46.8% | 0.00193 |
| McGill | T2T | deletion | 10 | 10 | dysgu | 91.9% | 91.9% | 0.00035 |
| McGill | T2T | insertion | 10 | 10 | dysgu | 94.1% | 94.1% | 0.00122 |
| McGill | T2T | deletion | 10 | 10 | sniffles2 | 92.2% | 92.2% | 0.00076 |
| McGill | T2T | insertion | 10 | 10 | sniffles2 | 92.8% | 92.8% | 0.00110 |
| McGill | T2T | deletion | 10 | 10 | svim | 93.5% | 93.5% | 0.00042 |
| McGill | T2T | insertion | 10 | 10 | svim | 95.1% | 95.1% | 0.00146 |
| McGill | T2T | deletion | 20 | 10 | cuteSV | 84.3% | 84.3% | 0.00066 |
| McGill | T2T | insertion | 20 | 10 | cuteSV | 87.2% | 87.2% | 0.00087 |
| McGill | T2T | deletion | 20 | 10 | dysgu | 93.8% | 93.8% | 0.00066 |
| McGill | T2T | insertion | 20 | 10 | dysgu | 95.0% | 95.0% | 0.00079 |
| McGill | T2T | deletion | 20 | 10 | sniffles2 | 94.2% | 94.2% | 0.00018 |
| McGill | T2T | insertion | 20 | 10 | sniffles2 | 95.0% | 95.0% | 0.00063 |
| McGill | T2T | deletion | 20 | 10 | svim | 95.3% | 95.3% | 0.00034 |
| McGill | T2T | insertion | 20 | 10 | svim | 96.4% | 96.3% | 0.00046 |
| McGill | T2T | deletion | 30 | 10 | cuteSV | 94.0% | 94.0% | 0.00017 |
| McGill | T2T | insertion | 30 | 10 | cuteSV | 95.3% | 95.3% | 0.00073 |
| McGill | T2T | deletion | 30 | 10 | dysgu | 94.2% | 94.2% | 0.00042 |
| McGill | T2T | insertion | 30 | 10 | dysgu | 94.9% | 94.9% | 0.00040 |
| McGill | T2T | deletion | 30 | 10 | sniffles2 | 94.6% | 94.6% | 0.00018 |
| McGill | T2T | insertion | 30 | 10 | sniffles2 | 95.4% | 95.4% | 0.00016 |
| McGill | T2T | deletion | 30 | 10 | svim | 95.2% | 95.2% | 0.00124 |
| McGill | T2T | insertion | 30 | 10 | svim | 96.5% | 96.5% | 0.00068 |
| NovaseqX | T2T | deletion | 5 | 10 | dysgu | 24.8% | 24.9% | 0.00405 |
| NovaseqX | T2T | insertion | 5 | 10 | dysgu | 4.9% | 4.9% | 0.00202 |
| NovaseqX | T2T | deletion | 10 | 10 | dysgu | 36.5% | 36.6% | 0.00317 |
| NovaseqX | T2T | insertion | 10 | 10 | dysgu | 9.4% | 9.4% | 0.00142 |
| NovaseqX | T2T | deletion | 20 | 10 | dysgu | 48.2% | 48.2% | 0.00138 |
| NovaseqX | T2T | insertion | 20 | 10 | dysgu | 15.5% | 15.5% | 0.00172 |
| NovaseqX | T2T | deletion | 30 | 9 | dysgu | 56.5% | 56.5% | 0.00109 |
| NovaseqX | T2T | insertion | 30 | 10 | dysgu | 21.0% | 21.1% | 0.00194 |
| SickKids | T2T | insertion | 5 | 10 | cuteSV | 0.2% | 0.2% | 0.00000 |
| SickKids | T2T | deletion | 5 | 10 | dysgu | 82.6% | 82.6% | 0.00143 |
| SickKids | T2T | insertion | 5 | 10 | dysgu | 87.1% | 87.1% | 0.00102 |
| SickKids | T2T | deletion | 5 | 10 | sniffles2 | 76.1% | 76.0% | 0.00137 |
| SickKids | T2T | insertion | 5 | 10 | sniffles2 | 77.3% | 77.3% | 0.00164 |
| SickKids | T2T | deletion | 5 | 10 | svim | 81.0% | 81.0% | 0.00098 |
| SickKids | T2T | insertion | 5 | 10 | svim | 86.4% | 86.4% | 0.00133 |
| SickKids | T2T | deletion | 10 | 10 | cuteSV | 51.3% | 51.3% | 0.00158 |
| SickKids | T2T | insertion | 10 | 10 | cuteSV | 53.8% | 53.7% | 0.00190 |
| SickKids | T2T | deletion | 10 | 10 | dysgu | 92.3% | 92.4% | 0.00080 |
| SickKids | T2T | insertion | 10 | 10 | dysgu | 94.7% | 94.7% | 0.00082 |
| SickKids | T2T | deletion | 10 | 10 | sniffles2 | 92.7% | 92.7% | 0.00040 |
| SickKids | T2T | insertion | 10 | 10 | sniffles2 | 93.8% | 93.8% | 0.00042 |
| SickKids | T2T | deletion | 10 | 10 | svim | 93.7% | 93.7% | 0.00052 |
| SickKids | T2T | insertion | 10 | 10 | svim | 95.5% | 95.5% | 0.00052 |
| SickKids | T2T | deletion | 20 | 10 | cuteSV | 84.2% | 84.1% | 0.00062 |
| SickKids | T2T | insertion | 20 | 10 | cuteSV | 88.3% | 88.3% | 0.00106 |
| SickKids | T2T | deletion | 20 | 10 | dysgu | 93.9% | 93.9% | 0.00031 |
| SickKids | T2T | insertion | 20 | 10 | dysgu | 95.5% | 95.5% | 0.00077 |
| SickKids | T2T | deletion | 20 | 10 | sniffles2 | 94.4% | 94.4% | 0.00031 |
| SickKids | T2T | insertion | 20 | 10 | sniffles2 | 95.8% | 95.8% | 0.00095 |
| SickKids | T2T | deletion | 20 | 10 | svim | 95.1% | 95.1% | 0.00090 |
| SickKids | T2T | insertion | 20 | 10 | svim | 96.4% | 96.4% | 0.00031 |
| SickKids | T2T | deletion | 30 | 10 | cuteSV | 94.1% | 94.1% | 0.00022 |
| SickKids | T2T | insertion | 30 | 10 | cuteSV | 95.6% | 95.6% | 0.00021 |
| SickKids | T2T | deletion | 30 | 10 | dysgu | 94.3% | 94.3% | 0.00037 |
| SickKids | T2T | insertion | 30 | 10 | dysgu | 95.5% | 95.5% | 0.00064 |
| SickKids | T2T | deletion | 30 | 10 | sniffles2 | 94.8% | 94.8% | 0.00033 |
| SickKids | T2T | insertion | 30 | 10 | sniffles2 | 95.9% | 95.9% | 0.00014 |
| SickKids | T2T | deletion | 30 | 10 | svim | 95.1% | 95.1% | 0.00044 |
| SickKids | T2T | insertion | 30 | 10 | svim | 96.8% | 96.8% | 0.00059 |
| BCGSC | CMRG | deletion | 5 | 10 | dysgu | 99.4% | 100.0% | 0.01860 |
| BCGSC | CMRG | insertion | 5 | 10 | dysgu | 84.9% | 85.0% | 0.01611 |
| BCGSC | CMRG | deletion | 5 | 10 | sniffles2 | 85.8% | 87.5% | 0.03553 |
| BCGSC | CMRG | insertion | 5 | 10 | sniffles2 | 71.9% | 71.9% | 0.02459 |
| BCGSC | CMRG | deletion | 5 | 10 | svim | 81.3% | 81.4% | 0.01199 |
| BCGSC | CMRG | insertion | 5 | 10 | svim | 88.9% | 88.9% | 0.00566 |
| BCGSC | CMRG | deletion | 10 | 10 | cuteSV | 64.2% | 66.7% | 0.04025 |
| BCGSC | CMRG | insertion | 10 | 2 | cuteSV | 0.0% | 0.0% | 0.00000 |
| BCGSC | CMRG | deletion | 10 | 10 | dysgu | 94.9% | 96.3% | 0.05349 |
| BCGSC | CMRG | insertion | 10 | 10 | dysgu | 93.7% | 93.8% | 0.01077 |
| BCGSC | CMRG | deletion | 10 | 10 | sniffles2 | 96.8% | 97.1% | 0.01235 |
| BCGSC | CMRG | insertion | 10 | 10 | sniffles2 | 93.0% | 93.0% | 0.01453 |
| BCGSC | CMRG | deletion | 10 | 10 | svim | 95.7% | 96.2% | 0.01272 |
| BCGSC | CMRG | insertion | 10 | 10 | svim | 93.0% | 93.0% | 0.00989 |
| BCGSC | CMRG | deletion | 20 | 10 | cuteSV | 89.8% | 88.9% | 0.01989 |
| BCGSC | CMRG | insertion | 20 | 10 | cuteSV | 89.9% | 89.9% | 0.00000 |
| BCGSC | CMRG | deletion | 20 | 10 | dysgu | 96.3% | 96.3% | 0.00000 |
| BCGSC | CMRG | insertion | 20 | 10 | dysgu | 97.5% | 97.3% | 0.00665 |
| BCGSC | CMRG | deletion | 20 | 10 | sniffles2 | 97.1% | 97.1% | 0.00000 |
| BCGSC | CMRG | insertion | 20 | 10 | sniffles2 | 95.0% | 95.0% | 0.00041 |
| BCGSC | CMRG | deletion | 20 | 10 | svim | 100.0% | 100.0% | 0.00000 |
| BCGSC | CMRG | insertion | 20 | 10 | svim | 95.8% | 95.7% | 0.00523 |
| BCGSC | CMRG | deletion | 30 | 10 | cuteSV | 100.0% | 100.0% | 0.00000 |
| BCGSC | CMRG | insertion | 30 | 10 | cuteSV | 97.3% | 97.3% | 0.00000 |
| BCGSC | CMRG | deletion | 30 | 10 | dysgu | 100.0% | 100.0% | 0.00000 |
| BCGSC | CMRG | insertion | 30 | 10 | dysgu | 96.1% | 95.3% | 0.01618 |
| BCGSC | CMRG | deletion | 30 | 10 | sniffles2 | 100.0% | 100.0% | 0.00000 |
| BCGSC | CMRG | insertion | 30 | 10 | sniffles2 | 95.1% | 95.1% | 0.00000 |
| BCGSC | CMRG | deletion | 30 | 10 | svim | 100.0% | 100.0% | 0.00000 |
| BCGSC | CMRG | insertion | 30 | 10 | svim | 95.4% | 95.5% | 0.00054 |
| McGill | CMRG | deletion | 5 | 10 | dysgu | 87.5% | 87.5% | 0.00000 |
| McGill | CMRG | insertion | 5 | 10 | dysgu | 89.4% | 89.8% | 0.01436 |
| McGill | CMRG | deletion | 5 | 10 | sniffles2 | 85.7% | 85.7% | 0.00000 |
| McGill | CMRG | insertion | 5 | 10 | sniffles2 | 76.5% | 76.2% | 0.01344 |
| McGill | CMRG | deletion | 5 | 10 | svim | 79.2% | 79.5% | 0.01210 |
| McGill | CMRG | insertion | 5 | 10 | svim | 84.0% | 83.7% | 0.01130 |
| McGill | CMRG | deletion | 10 | 10 | cuteSV | 58.8% | 58.8% | 0.00000 |
| McGill | CMRG | insertion | 10 | 10 | cuteSV | 43.2% | 43.0% | 0.05889 |
| McGill | CMRG | deletion | 10 | 10 | dysgu | 98.6% | 98.6% | 0.01424 |
| McGill | CMRG | insertion | 10 | 10 | dysgu | 94.4% | 94.3% | 0.00651 |
| McGill | CMRG | deletion | 10 | 10 | sniffles2 | 94.6% | 95.7% | 0.01616 |
| McGill | CMRG | insertion | 10 | 10 | sniffles2 | 93.6% | 93.7% | 0.00299 |
| McGill | CMRG | deletion | 10 | 10 | svim | 96.7% | 96.3% | 0.01171 |
| McGill | CMRG | insertion | 10 | 10 | svim | 94.9% | 94.9% | 0.00156 |
| McGill | CMRG | deletion | 20 | 10 | cuteSV | 81.4% | 82.4% | 0.06111 |
| McGill | CMRG | insertion | 20 | 10 | cuteSV | 91.9% | 91.9% | 0.01920 |
| McGill | CMRG | deletion | 20 | 10 | dysgu | 100.0% | 100.0% | 0.00000 |
| McGill | CMRG | insertion | 20 | 10 | dysgu | 94.5% | 94.5% | 0.00021 |
| McGill | CMRG | deletion | 20 | 10 | sniffles2 | 92.0% | 92.0% | 0.00338 |
| McGill | CMRG | insertion | 20 | 10 | sniffles2 | 94.9% | 94.9% | 0.00000 |
| McGill | CMRG | deletion | 20 | 10 | svim | 100.0% | 100.0% | 0.00000 |
| McGill | CMRG | insertion | 20 | 10 | svim | 95.9% | 96.3% | 0.00546 |
| McGill | CMRG | deletion | 30 | 10 | cuteSV | 85.7% | 85.7% | 0.00000 |
| McGill | CMRG | insertion | 30 | 10 | cuteSV | 97.3% | 97.3% | 0.00000 |
| McGill | CMRG | deletion | 30 | 10 | dysgu | 100.0% | 100.0% | 0.00000 |
| McGill | CMRG | insertion | 30 | 10 | dysgu | 92.7% | 92.7% | 0.00000 |
| McGill | CMRG | deletion | 30 | 10 | sniffles2 | 97.1% | 97.1% | 0.00000 |
| McGill | CMRG | insertion | 30 | 10 | sniffles2 | 94.0% | 94.0% | 0.00000 |
| McGill | CMRG | deletion | 30 | 10 | svim | 100.0% | 100.0% | 0.00000 |
| McGill | CMRG | insertion | 30 | 10 | svim | 95.2% | 95.2% | 0.00000 |
| NovaseqX | CMRG | deletion | 5 | 10 | dysgu | 27.0% | 27.2% | 0.02694 |
| NovaseqX | CMRG | insertion | 5 | 10 | dysgu | 11.3% | 10.1% | 0.02254 |
| NovaseqX | CMRG | deletion | 10 | 10 | dysgu | 37.7% | 37.9% | 0.01582 |
| NovaseqX | CMRG | insertion | 10 | 10 | dysgu | 16.3% | 15.9% | 0.01749 |
| NovaseqX | CMRG | deletion | 20 | 10 | dysgu | 50.4% | 50.6% | 0.01276 |
| NovaseqX | CMRG | insertion | 20 | 10 | dysgu | 20.0% | 20.3% | 0.01614 |
| NovaseqX | CMRG | deletion | 30 | 10 | dysgu | 58.6% | 58.6% | 0.01001 |
| NovaseqX | CMRG | insertion | 30 | 10 | dysgu | 22.9% | 22.8% | 0.01102 |
| SickKids | CMRG | deletion | 5 | 10 | dysgu | 82.2% | 83.3% | 0.04648 |
| SickKids | CMRG | insertion | 5 | 10 | dysgu | 89.8% | 89.7% | 0.02119 |
| SickKids | CMRG | deletion | 5 | 10 | sniffles2 | 81.0% | 80.9% | 0.03466 |
| SickKids | CMRG | insertion | 5 | 10 | sniffles2 | 79.7% | 79.2% | 0.02235 |
| SickKids | CMRG | deletion | 5 | 10 | svim | 85.9% | 85.9% | 0.00631 |
| SickKids | CMRG | insertion | 5 | 10 | svim | 87.6% | 87.7% | 0.00686 |
| SickKids | CMRG | deletion | 10 | 10 | cuteSV | 65.3% | 65.3% | 0.01405 |
| SickKids | CMRG | insertion | 10 | 10 | cuteSV | 54.5% | 55.5% | 0.06266 |
| SickKids | CMRG | deletion | 10 | 10 | dysgu | 95.3% | 100.0% | 0.08183 |
| SickKids | CMRG | insertion | 10 | 10 | dysgu | 95.4% | 95.3% | 0.00697 |
| SickKids | CMRG | deletion | 10 | 10 | sniffles2 | 93.2% | 94.1% | 0.01699 |
| SickKids | CMRG | insertion | 10 | 10 | sniffles2 | 93.9% | 94.2% | 0.01154 |
| SickKids | CMRG | deletion | 10 | 10 | svim | 97.6% | 97.6% | 0.01544 |
| SickKids | CMRG | insertion | 10 | 10 | svim | 93.9% | 94.3% | 0.00986 |
| SickKids | CMRG | deletion | 20 | 10 | cuteSV | 88.8% | 87.8% | 0.01517 |
| SickKids | CMRG | insertion | 20 | 10 | cuteSV | 83.8% | 83.8% | 0.00000 |
| SickKids | CMRG | deletion | 20 | 10 | dysgu | 96.3% | 96.3% | 0.00000 |
| SickKids | CMRG | insertion | 20 | 10 | dysgu | 95.8% | 95.8% | 0.00430 |
| SickKids | CMRG | deletion | 20 | 10 | sniffles2 | 100.0% | 100.0% | 0.00000 |
| SickKids | CMRG | insertion | 20 | 10 | sniffles2 | 95.7% | 95.3% | 0.00484 |
| SickKids | CMRG | deletion | 20 | 10 | svim | 100.0% | 100.0% | 0.00000 |
| SickKids | CMRG | insertion | 20 | 10 | svim | 95.1% | 95.1% | 0.00156 |
| SickKids | CMRG | deletion | 30 | 10 | cuteSV | 100.0% | 100.0% | 0.00000 |
| SickKids | CMRG | insertion | 30 | 10 | cuteSV | 97.2% | 97.1% | 0.00080 |
| SickKids | CMRG | deletion | 30 | 10 | dysgu | 100.0% | 100.0% | 0.00000 |
| SickKids | CMRG | insertion | 30 | 10 | dysgu | 98.0% | 98.0% | 0.00000 |
| SickKids | CMRG | deletion | 30 | 10 | sniffles2 | 100.0% | 100.0% | 0.00000 |
| SickKids | CMRG | insertion | 30 | 10 | sniffles2 | 96.8% | 96.8% | 0.00000 |
| SickKids | CMRG | deletion | 30 | 10 | svim | 100.0% | 100.0% | 0.00000 |
| SickKids | CMRG | insertion | 30 | 10 | svim | 95.6% | 96.0% | 0.01005 |

**Statistics computed only on successfully completed Variant Calling Analyses*

**Supplementary Table 2: *De novo* Assembly QUAST-Derived Metrics (by Haplotype)**

|  | **BCGSC hap1** | **BCGSC hap2** | **McGill hap1** | **McGill hap2** | **SickKids hap1** | **SickKids hap2** |
| --- | --- | --- | --- | --- | --- | --- |
| *Genome fraction (%)* | 94.236 | 91.771 | 92.667 | 93.611 | 90.583 | 94.983 |
| *Duplication ratio* | 1.019 | 1.016 | 1.019 | 1.016 | 1.019 | 1.018 |
| *Largest alignment* | 145,203,959 | 145,455,560 | 110,884,220 | 110,883,412 | 144,786,502 | 111,660,202 |
| *Total aligned length* | 2,992,754,257 | 2,906,896,952 | 2,944,994,499 | 2,965,843,361 | 2,876,692,796 | 3,013,924,442 |
| *NGA50* | 50,070,319 | 59,360,335 | 37,685,606 | 39,702,711 | 40,198,398 | 42,075,534 |
| *LGA50* | 20 | 16 | 25 | 23 | 21 | 20 |
| **Per base quality** | | | | | | |
| *# mismatches per 100 kbp* | 136.6 | 131.13 | 138.07 | 133.5 | 139.43 | 135.91 |
| *# indels per 100 kbp* | 29.47 | 29.71 | 29.49 | 29.71 | 29.9 | 29.23 |
| *# N's per 100 kbp* | 0 | 0 | 0 | 0 | 0 | 0 |
| **Statistics without reference** | | | | | |  |
| *# contigs* | 206 | 191 | 298 | 258 | 240 | 224 |
| *Largest contig* | 160,339,046 | 145,475,625 | 201,505,653 | 139,528,439 | 192,341,870 | 200,915,923 |
| *Total length* | 3,026,179,206 | 2,938,913,227 | 2,978,512,720 | 3,003,095,313 | 2,913,695,489 | 3,048,901,211 |
| *Total length (>= 10000 bp)* | 3,026,174,986 | 2,938,913,227 | 2,978,435,734 | 3,003,086,184 | 2,913,695,489 | 3,048,901,211 |

## 1.2 Supplementary Figures

**Supplementary Figure 1: Small Germline Variant Detection Performance on the CMRG v1.0 Truth Set.**

Plot comparing the SNV (top row) and Indel (bottom row) calls to the CMRG v1.0, [which includes challenging medically relevant genes] truth set and the resulting F1, Precision, and Recall scores for the three ONT datasets, as well as the results of an *Illumina* NovaSeqX dataset of the same cell-line sequenced at mean depth of 46.4× and 315.8 bp mean insert size.

**Supplementary Figure 2: Small Variant Performance Segregated by Genomic Region**

Plot comparing the SNV (left column) and Indel (right column) germline small variants performance (F1 – top row, precision – middle row, and recall – bottom row) by the genomic context (i.e. difficult, RefSeq coding, homopolymers, etc.) using the classification developed by (Jarvis *et al.*, 2022).

**Supplementary Figure 3: Visualization of the Insertion in Gene GPI**

View of the known 16,946 bp insertion in an intronic VNTR in the gene GPI. The insertion can be observed in all the samples. The first 3 tracks correspond to the BCGSC sample, the next 3 tracks to the McGill sample and the following 3 tracks to the SickKids. Tracks colored in grey represent total depth of coverage; tracks in yellow are a pileup corresponding to haplotype 1 and tracks in magenta are a pileup corresponding to haplotype 2.

**Supplementary Figure 4: Visualization of a Phasing Block in all Samples.**

Graph genomes (**[A]** BCGSC, **[B]** McGill and **[C]** SickKids/TCAG) were split into 100 kb segments and each query sample was compared to both paternal and maternal HG002 Q100 sequences. Greater similarity to paternal haplotype is colored as blue and pink for maternal. A sliding window of 500 kb was used to smooth the plot. All assemblies contain a high degree of phase switching.

**Supplementary Figure 5: Performance of SNV/InDel Calling at Different Depths**

Line plot showing the difference in average F1 score for each sample at the downsampled depths of 5, 10, 20, and 30x using the T2T truth set. Each sample was randomly downsampled 10 times using different seeds to account for potential bias. Error bars indicate the standard deviation range (although at higher depths they become too small to properly distinguish). The top panel shows results for SNVs and the bottom panel for INDELs. Full values available in **Table 3.**

**Supplementary Figure 6: Performance of SV Callers at Different Depths**

Line plot showing the difference in average F1 score for each sample and SV caller at the downsampled depths of 5, 10, 20, and 30x using both the T2T SV truth set (**A**) and the CMRG SV truth set (**B**). Each sample was randomly downsampled 10 times using different seeds to account for potential bias. Error bars indicate the standard deviation range (although at higher depths they become too small to properly distinguish). The top panel shows results for deletions (del) and the bottom panel for insertions (ins). Full values available in **Table S1.**

**Supplementary Figure 7:** **Methylation calls on downsampled cross-centre benchmark data to 30×, 20× and 10×.**

Correlation coefficient matrix of the methylation percentage at each CpG site for samples produced at each sequencing centre at different down sampled depths: **(A)** 30×, **(B)** 20× and **(C)** 10×. The number of CpG sites interrogated is included in each box.

**Supplementary Figure 8: Difference in Methylation Between Haplotypes at Imprinting Regions.**

Difference in methylation between haplotypes at imprinting regions (iDMRs) across all three samples and all autosomal chromosomes.

**Supplementary Figure 9: Correlation of 5-hmC Calls Across Datasets.**

Correlation coefficient matrix of the 5-hmC percentage at each CpG site for samples produced at each sequencing centre. The number of CpG sites interrogated is included in each box.
